# First-line therapies for unresectable hepatocellular carcinoma: a network meta-analysis of immune checkpoint inhibitors and transarterial therapies based on 35 randomized trials

**DOI:** 10.3389/fonc.2026.1809232

**Published:** 2026-04-29

**Authors:** Huanghui Zhang, Shenyao Zhou, Jiaxin Dai, Hong Huang, Bing Yang, Wenqi Huang, Dongxin Tang

**Affiliations:** 1 Guizhou University of Traditional Chinese Medicine, Guiyang, China; 2 Faculty of Chinese Medicine Science Guangxi University of Chinese Medicine, Nanning, China; 3 First Affiliated Hospital of Guizhou University of Traditional Chinese Medicine, Guiyang, China; 4 Affiliated Hospital of Zunyi Medical University, Zunyi, China

**Keywords:** first-line treatment, hepatocellular carcinoma, immune checkpoint inhibitors, network meta-analysis, transarterial therapies

## Abstract

**Objective:**

Unresectable hepatocellular carcinoma (uHCC) is a major global health challenge. Current first-line strategies involve immune checkpoint inhibitors (ICIs), transarterial therapies, and anti-VEGF agents. However, comprehensive comparative efficacy and safety, including certainty assessments, for diverse combination interventions are lacking. This study systematically compared 30 first-line uHCC interventions using network meta-analysis, integrating evidence certainty, to inform clinical decisions and refine guideline recommendations.

**Methods:**

Following a preregistered protocol (PROSPERO: CRD420261303389), a systematic search of major databases (until Jan 10, 2026) identified randomized controlled trials (RCTs) on first-line uHCC monotherapies or combinations (ICIs, transarterial therapies). Primary outcomes were overall survival (OS) and progression-free survival (PFS); secondary outcomes included objective response rate (ORR) and grade ≥3 adverse events (AEs). Screening, data extraction, and risk of bias (Cochrane RoB 2.0) were conducted by two reviewers. A Bayesian random-effects network meta-analysis, with certainty of evidence assessed by CINeMA and GRADE, synthesized direct and indirect evidence, reporting HRs/ORs (95% CrIs) and ranking interventions (SUCRA).

**Results:**

Thirty-five RCTs (13,595 participants, 30 interventions) were analyzed. Most studies had low bias risk; overall evidence certainty was moderate. HAIC-Sora significantly improved OS (HR 0.57; 95% CrI 0.36-0.89; moderate certainty) and PFS (HR 0.46; 95% CrI 0.28-0.75; moderate certainty) versus sorafenib. HAIC notably improved ORR (OR 29.94; 95% CrI 3.38-265.11, high certainty). TACE-Lenv significantly reduced grade ≥3 AEs (OR 0.23; 95% CrI 0.06-0.87; moderate certainty) versus sorafenib.

**Conclusion:**

For uHCC patients, HAIC-Sora demonstrated superior OS and PFS with an acceptable safety profile, representing the only comparison achieving statistical significance against sorafenib monotherapy for survival outcomes. While SUCRA rankings provided a relative hierarchy of other treatments, their interpretation should be approached with caution due to predominantly indirect evidence and lack of statistical significance in most pairwise comparisons. Limitations include blinding challenges and a reliance on indirect evidence for many comparisons, contributing to moderate evidence certainty. High-quality future studies, especially head-to-head comparisons, are crucial to solidify findings and guide clinical practice.

**Systematic Review Registration:**

https://www.crd.york.ac.uk/PROSPERO/, identifier CRD420261303389.

## 1 Introduction

According to GLOBOCAN 2022 statistics, primary liver cancer ranks as the sixth most common cancer globally, with an estimated 865,269 new cases annually, and stands as the third leading cause of cancer-related mortality, accounting for approximately 757,948 fatalities each year (1). This exceptionally high mortality-to-incidence ratio (MIR) underscores the substantial public health challenge presented by the disease, often characterized by late diagnosis and a dismal prognosis. Furthermore, primary liver cancer demonstrates marked geographical heterogeneity, with particularly elevated incidence rates observed in transition economies and regions with a medium to high Human Development Index. The disease burden is particularly acute in East Asia, notably Mongolia and China, Southeast Asia, parts of North and West Africa, and certain regions of Central America. A significant sex disparity is also evident, with primary liver cancer ranking as the second leading cause of cancer-related death in males, whereas it does not feature among the top five for females (1).

Hepatocellular carcinoma (HCC) is the predominant form of primary liver cancer, accounting for approximately 75% to 85% of cases. Major risk factors for HCC include chronic viral hepatitis (caused by HBV or HCV), aflatoxin exposure, smoking, alcohol consumption, obesity, and type 2 diabetes mellitus. These factors contribute to chronic liver diseases, such as alcoholic liver disease (ALD) and non-alcoholic fatty liver disease (NAFLD), which are key drivers of HCC development (1). The overall prognosis for HCC remains exceptionally poor, with a 5-year survival rate of less than 20% (2). Unfortunately, 70% to 80% of patients are diagnosed at advanced or unresectable stages, precluding curative surgical intervention and resulting in a mere 3% 5-year survival rate (3). Projections suggest that by 2040, global liver cancer incidence will exceed 1.4 million cases, with deaths reaching 1.3 million (4). Consequently, the imperative to develop more effective treatment strategies is pressing.

The advent of ICIs has significantly transformed first-line treatment paradigms for advanced HCC in recent years. Historically, anti-vascular endothelial growth factor (anti-VEGF) agents like sorafenib and lenvatinib constituted the standard first-line therapies; however, their use was often limited by modest efficacy, a high incidence of AEs, and a propensity for developing resistance. Following the IMbrave150 trial (5), which demonstrated the superiority of atezolizumab plus bevacizumab over sorafenib, ICIs and their combination regimens have rapidly entered clinical practice (6). ICIs are monoclonal antibodies specifically designed to target immune checkpoint receptors on the surface of T cells. Key drugs in this class include pembrolizumab, nivolumab, tislelizumab, and camrelizumab (7). The primary targets of ICIs include the PD-1/PD-L1 and CTLA-4 pathways, which tumor cells exploit within the tumor microenvironment to suppress T-cell activity, thereby facilitating immune evasion (8). Their mechanism of action involves binding to these targets, such as PD-1, PD-L1, and CTLA-4, thereby disinhibiting immunosuppressive signals and restoring the T-cell-mediated cytotoxic effect against tumor cells (9). Several clinical trials, including RATIONALE-301 and CheckMate459, have consistently shown that, compared with anti-VEGF therapies, ICIs significantly extend PFS while being associated with a significantly lower incidence of AEs (10, 11).

Transarterial therapies represent crucial local treatment modalities for HCC, primarily encompassing transarterial chemoembolization (TACE) and hepatic arterial infusion chemotherapy (HAIC) (12). TACE exerts its therapeutic effect through the occlusion of tumor-feeding arteries, inducing tumor necrosis via ischemia and hypoxia, and synergistically inhibiting and eliminating tumor cells when combined with co-administered chemotherapeutic agents. Numerous international clinical guidelines recommend TACE as a primary treatment for unresectable HCC, recognizing its capacity to significantly improve survival in these patients (13). HAIC, in contrast, involves percutaneous catheter placement into the hepatic artery proper and its branches for prolonged, continuous infusion of chemotherapeutic agents to eradicate tumor cells. Compared with systemic intravenous chemotherapy, HAIC significantly increases local drug concentrations within the liver, ensuring sustained tumor drug exposure while minimizing damage to healthy liver tissue and reducing systemic adverse effects. Clinical trials have demonstrated HAIC’s capacity to significantly improve OS compared with TACE (14). However, both treatments are associated with notable AEs. Post-embolization syndrome (characterized by fever, hepatic pain, nausea, and vomiting) is most common after TACE, while HAIC can lead to catheter-related complications such as infection, hepatotoxicity, and gastrointestinal reactions. Most of these AEs are manageable with supportive and symptomatic treatment (15). Although HAIC has emerged as a promising treatment approach for HCC in recent years, guidelines from organizations such as the National Comprehensive Cancer Network (NCCN) and the European Association for the Study of the Liver (EASL) have not yet incorporated HAIC as a routine recommendation for advanced HCC. In contrast, HAIC is recognized as an effective treatment modality for intermediate to advanced HCC and is included in guidelines in several Asian countries (16).

Owing to the challenges posed by late diagnosis, aggressive disease biology, and inherent drug resistance, monotherapy for HCC frequently yields suboptimal clinical outcomes. Consequently, exploring dual or triple combination regimens involving anti-VEGF agents, ICIs, and transarterial therapies (TACE, HAIC) has emerged as a crucial new therapeutic direction. Recent pivotal trials have already underscored the synergistic benefits of these dual and triple regimens. For instance, the LEAP-012 trial demonstrated that the triple combination of lenvatinib, pembrolizumab, and TACE showed superiority over TACE monotherapy in terms of both PFS and OS (17). Similarly, the TALENTACE trial investigated the efficacy of TACE plus atezolizumab and bevacizumab, reporting a significant improvement in PFS with a safety profile comparable to existing regimens (18). The EMERALD-1 trial, which directly compared the triple regimen of TACE + durvalumab + bevacizumab, the dual regimen of TACE + durvalumab, and TACE monotherapy, provided compelling evidence supporting the clinical utility of triple therapy (19). Reflecting this accumulating evidence, TACE plus anti-VEGF plus ICIs is now incorporated into Class I recommendations for stage IIb and IIIa HCC in the Chinese Society of Clinical Oncology (CSCO) guidelines (20).

The accumulating body of clinical trial data and evolving guideline updates unmistakably highlight the promising prospects of combination therapies involving anti-VEGF agents, ICIs, and transarterial interventions such as TACE and HAIC. However, the absence of direct head-to-head comparisons among these diverse regimens underscores an unmet need for network meta-analysis to provide high-level evidence that can guide optimal therapeutic sequencing and selection (21). To address this gap, the current network meta-analysis synthesized existing randomized controlled trial data to comprehensively evaluate the efficacy and safety of both monotherapies and combination regimens involving ICIs or transarterial therapies in the management of uHCC. Our analysis specifically focused on comparing outcomes across key survival endpoints (OS and PFS), ORR, and safety profiles (AEs), aiming to provide robust evidence to inform first-line treatment decisions for uHCC.

## 2 Materials and methods

This network meta-analysis was conducted in accordance with the Preferred Reporting Items for Systematic Reviews and Meta-Analysis extension statement for network meta-analyses (PRISMA-NMA) (**Supplementary Table 1**) (22). For transparency and reliability, the study protocol was prospectively registered with the International Prospective Register of Systematic Reviews (PROSPERO: CRD420261303389).

### 2.1 Data sources and search strategy

We systematically searched PubMed, EMBASE, Cochrane Library, and Web of Science databases. The search strategy combined free-text terms with controlled vocabulary (MeSH terms/Embase controlled vocabulary). Key search terms encompassed, but were not limited to, “Hepatocellular Carcinoma,” “Randomized controlled trial,” “Immune checkpoint inhibitors,” “PD-L1 inhibitor,” “PD-1 inhibitor,” “CTLA-4 Inhibitor,” “Pembrolizumab,” “Camrelizumab,” “Nivolumab,” “Ipilimumab,” “Transarterial chemoembolization,” and “Hepatic arterial infusion chemotherapy” (the full search strategy is detailed in **Supplementary Table 2**). The search spanned from each database’s inception until January 10, 2026, and was conducted without language restrictions.

### 2.2 Selection criteria

Inclusion Criteria: RCTs were included if they met the following conditions (1): RCTs enrolling patients with histologically or cytologically confirmed uHCC. Patients aged 18 years or older, currently undergoing first-line therapy, were included if they had Child-Pugh Class A or B liver function prior to treatment initiation and no severe, uncontrolled comorbidities, such as severe renal or cardiac dysfunction, active infection, or autoimmune diseases requiring systemic immunosuppression (2). RCTs investigating monotherapies or combination regimens involving ICIs or transarterial therapies as first-line interventions for uHCC (3). RCTs comparing monotherapies or combination regimens involving ICIs or transarterial therapies to other treatment regimens for uHCC (4). RCTs reporting on at least one of the following outcome measures: (a) OS, defined as the time from randomization to death from any cause; (b) PFS, defined as the time from randomization to disease progression or death from any cause; (c) ORR, defined as the proportion of patients achieving an objective response (complete response or partial response); (d) AEs of grade ≥3 as defined by the National Cancer Institute Common Terminology Criteria for Adverse Events (CTCAE).

Exclusion Criteria: Studies were excluded if they met any of the following criteria (1): Studies presenting duplicate data from the same patient cohort (e.g., interim analyses or subsequent reports with overlapping data) (2). RCTs with unclear or insufficiently reported outcome measures (3). Review articles or case reports.

Initially, studies were screened by title and abstract. Subsequently, all potentially relevant RCTs underwent full-text review. Two independent reviewers (ZHH and ZSY) performed this screening to ensure accurate and consistent identification of eligible studies. Any discrepancies were resolved by discussion with a third author (DJX).

### 2.3 Data extraction and quality assessment

Data extraction from eligible RCTs was performed independently by two investigators (ZHH and ZSY) in accordance with the Preferred Reporting Items for Systematic Reviews and Meta-Analyses (PRISMA) guidelines. Discrepancies were resolved through discussion with a third author (DJX). From each included article, we extracted the following information: trial name, NCT number, first author, publication year, study phase, sample size, patient demographics (age, sex distribution), treatment regimens (experimental and control arms), and baseline disease characteristics (e.g., BCLC stage, Child-Pugh class, ECOG performance status, HBV/HCV/non-viral etiology, ALBI score, AFP ≥400 ng/mL, presence of extrahepatic metastasis, portal vein tumor thrombosis, macroscopic vascular invasion, and portal vein invasion). For outcomes, we extracted HRs with their 95% CrIs for OS and PFS, and ORs with 95% CrIs for ORR and grade ≥3 AEs.

The methodological quality of included RCTs was assessed using the Cochrane Risk of Bias Tool (RoB 2.0) (23). This tool evaluates RCTs across five domains: risk of bias arising from the randomization process, risk of bias due to deviations from intended interventions, risk of bias from missing outcome data, risk of bias in the measurement of the outcome, and risk of bias in the selection of the reported result. The risk of bias for each domain, as well as the overall risk of bias, was categorized as “low risk,” “some concerns,” or “high risk.”.

### 2.4 Statistical analysis

OS and PFS served as the primary outcomes, while ORR and grade ≥3 AEs were considered secondary outcomes. HRs with their 95% CrIs were employed as the effect measures for OS and PFS. For ORR and grade ≥3 AEs, ORs with their 95% CrIs were utilized. For a comprehensive overview of the detailed quantitative outcomes, including OS, PFS, ORR, and grade ≥3 AEs for all included randomized controlled trials, please refer to **Supplementary Table 6**.

The network meta-analysis was performed within a Bayesian framework, using the gemtc and rjags packages within R software. A random-effects model was selected as the primary analytical model to yield more conservative efficacy estimates, given the anticipated clinical heterogeneity across the included RCTs concerning population characteristics, geographical regions, and intervention specifics. Model fitting was conducted using Markov Chain Monte Carlo (MCMC) simulation, generating four independent Markov chains. Each chain underwent 15,000 burn-in iterations to facilitate convergence and eliminate the influence of initial values, followed by 100,000 sampling iterations to derive stable posterior distribution estimates. Model convergence was assessed comprehensively using the Gelman-Rubin diagnostic statistic and visual inspection of trace plots.

The ranking of interventions for efficacy and safety was determined using Surface Under the Cumulative RAnking Curve (SUCRA) values, with values closer to 1 indicating a superior intervention. Furthermore, probabilities for each intervention to achieve specific rank positions were derived from the Bayesian posterior distributions. The rank.probability() function extracted the probability matrix, and a heatmap, generated using the pheatmap function, visually represented the relative performance of interventions.

Model consistency was evaluated at both global and local levels. Global inconsistency was assessed by comparing the Deviance Information Criterion (DIC) values between the consistency and inconsistency models; a difference in DIC values greater than 5 was indicative of significant global inconsistency. Local inconsistency was investigated via the node-splitting method to identify discrepancies between direct and indirect evidence, where a P-value less than 0.05 suggested local inconsistency. To confirm the robustness of our findings, a fixed-effects model was also fitted as a sensitivity analysis. If the DIC difference between the random-effects and fixed-effects models was less than 5, the results were considered robust. Finally, comparison-adjusted funnel plots were constructed using STATA 17.0 software to evaluate potential publication bias and small study effects.

## 3 Results

### 3.1 Systematic review and characteristics of the included studies

**Figure 1:**
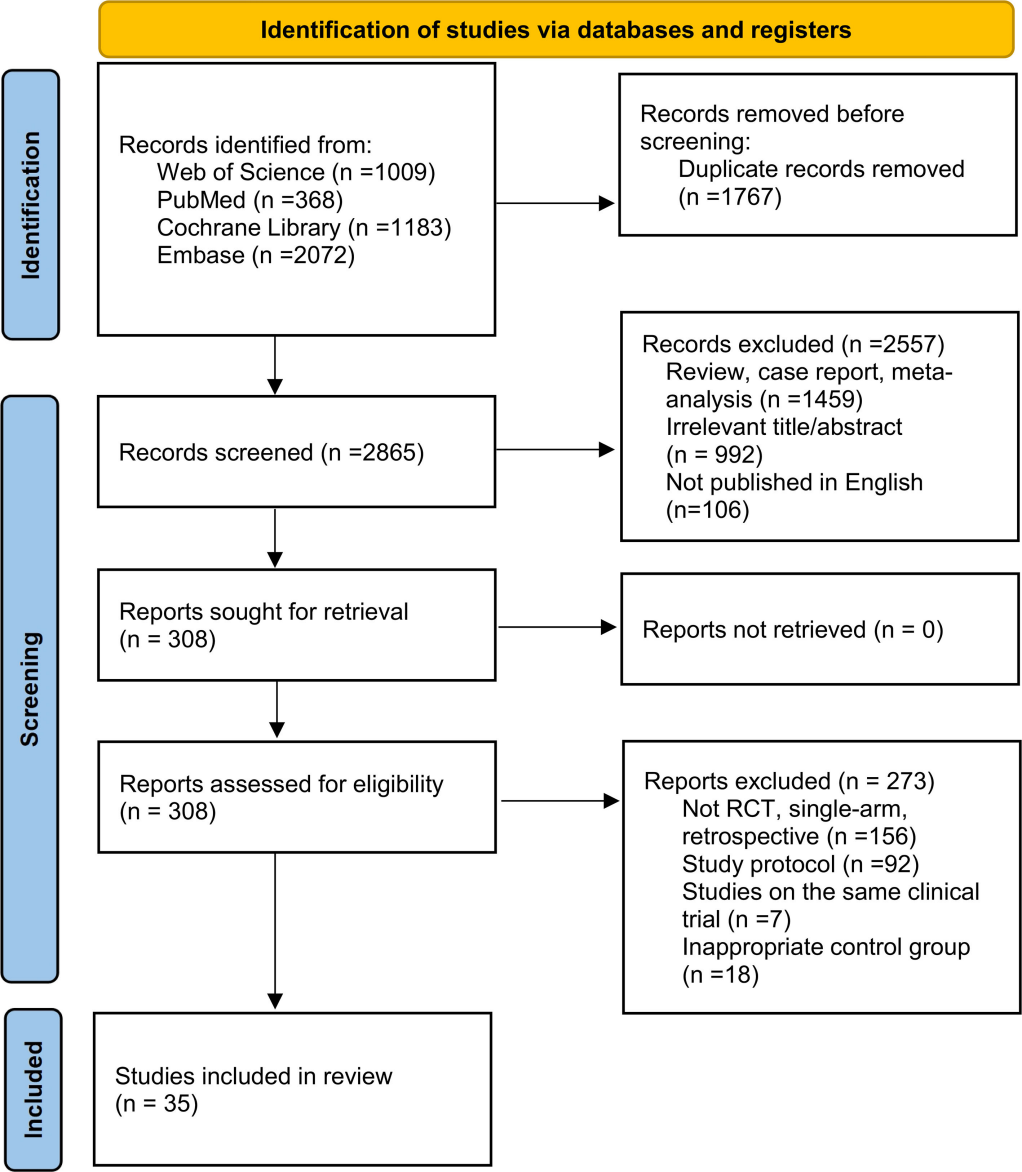
Flowchart according to the preferred reporting items for systematic reviews and meta analyses (PRISMA) guideline.

The initial literature search identified 4,632 records. Following abstract screening and removal of duplicates and irrelevant articles, 308 studies were selected for full-text review. Ultimately, 35 studies satisfied our predefined eligibility criteria (**Figure 1**). Comprehensive details of the included studies are provided in **Table 1** and **Table 2**.

**Table 1:** Baseline characteristics of studies included in the network meta-analysis (1).

Study	Study phase	Sample size	Median age	Male(%)	Geographical region (%)	Intervention arm	Control arm	Reported outcomes	Timing of data cut-off
Chen 2025 (24)	3	25/11	-	-/-	China (100.0)	Radiotherapy 40–60 Gy in 10 fractions + Toripalimab 240 mg Q3W	Sorafenib 400 mg BID	OS, ORR	December 27, 2024
Cheng 2022 (5) (IMbrave150)	3	336/165	–	277(82.4)/ 137(83.0)	Asia (40.1), Japan and others (59.9)	Atezolizumab 1200 mg Q3W + bevacizumab 15 mg/kg Q3W	Sorafenib 400 mg BID	OS, PFS, ORR, AEs	August 31, 2020
Ding 2021 (25)	-	32/32	57/56	25(78.1)/ 27(84.4)	China (100.0)	TACE + lenvatinib 8/12 mg QD	TACE + sorafenib 400 mg BID	OS, ORR, AEs	-
Dong 2025 (18) (TALENTACE)	3	171/171	–	-/-	China and Japan (100.0)	TACE + atezolizumab 1200 mg Q3W + bevacizumab 15 mg/kg Q3W	TACE	PFS, AEs	–
Duan 2024 (26)	3	122/121	57.5/58.8	100(82.0)/ 107(88.4)	China (100.0)	DEB-TACE + apatinib 500 mg QD	DEB-TACE	OS, PFS	July 01, 2023
Finn 2025 (27) (IMbrave152)	3	331/338	–	-/-	Global (100.0)	Tiragolumab 600 mg Q3W + atezolizumab 1200 mg Q3W + bevacizumab 15 mg/kg Q3W	Placebo + Atezolizumab 1200 mg Q3W + bevacizumab 15 mg/kg Q3W	OS, PFS, ORR, AEs	May 08, 2025
Finn 2025 (28) (MORPHEUS -Liver)	1b/2	40/18	65.5/62	9(22.5)/ 3(16.7)	Asia (25.9), Others (74.1)	Tiragolumab 600 mg Q3W + atezolizumab 1200 mg Q3W + bevacizumab 15 mg/kg Q3W	Atezolizumab 1200 mg Q3W + bevacizumab 15 mg/kg Q3W	OS, PFS, ORR, AEs	August 21, 2023
He 2019 (15) (SoraHAIC)	-	125/122	49/49	111(88.8)/ 112(91.8)	China (100.0)	Sorafenib 400 mg BID + HAIC (oxaliplatin 85 mg/m2, leucovorin 400 mg/m2, fluorouracil bolus 400 mg/m2 on day 1, and fluorouracil 2400 mg/m2 for 46 hours, Q3W)	Sorafenib 400 mg BID	OS, PFS, ORR, AEs	August 06, 2018
Ikeda 2016 (29)	2	65/41	66/64	56(86.2)/ 32(78.0)	Japan (100.0)	Sorafenib 400 mg BID + HAIC (cisplatin 65 mg/m2 Q4-6W)	Sorafenib 400 mg BID	OS, PFS, ORR	December 28, 2014
Kondo 2019 (30) (SCOOP-2)	2	35/33	72/70.9	28(80.0)/ 27(81.8)	Japan (100.0)	HAIC (cisplatin 65 mg/m2 Q4-6W) + Sorafenib 400 mg BID	Sorafenib 400 mg BID	OS, ORR	October, 2015
Kudo 2018 (31) (SILIUS)	3	102/103	69/68	89(87.3)/ 88(85.4)	Japan (100.0)	Sorafenib 400 mg BID + HAIC (cisplatin 20 mg/m2 on days 1 and 8, fluorouracil 330 mg/m2 continuously on days 1–5 and 8–12, Q3W)	Sorafenib 400 mg BID	OS, PFS, ORR	-
Kudo 2018 (32) (REFLECT)	3	478/476	63/62	405(84.7)/ 401(84.2)	Asia-Pacific (67.1), Western (32.9)	Lenvatinib 8/12 mg QD	Sorafenib 400 mg BID	OS, PFS, ORR, AEs	November 13, 2016
Kudo 2022 (33) (TACTICS)	2	80/76	72/73	63(78.8)/ 55(72.4)	Japan (100.0)	TACE + sorafenib 400 mg BID	TACE	OS, PFS	July 31, 2020
Kudo 2024 (17) (LEAP-012)	3	237/243	65/66	192(81.0)/ 206(84.8)	Asia (56.7), Japan and others (43.3)	Lenvatinib 8/12 mg QD + pembrolizumab 400 mg Q6W + TACE	Placebo + TACE	OS, PFS, ORR, AEs	January 30, 2024
Lai 2025 (34) (SHATA-001)	3	141/66	50/50.5	127(90.1)/ 57(86.4)	China (100.0)	Sorafenib 400 mg BID + FOLFOX-HAIC (oxaliplatin 85 mg/m2, leucovorin 400 mg/m2, fluorouracil 400 mg/m2, and fluorouracil 2400 mg/m2 Q3W)	Sorafenib 400 mg BID + TACE	OS, PFS, ORR, AEs	May 01, 2022
Lau 2025 (35) (HIMALAYA)	3	393/389	65/64	327(83.2)/ 337(86.6)	Asia (40.9), Japan and others (59.1)	Tremelimumab 300 mg single dose + durvalumab 1500 mg Q4W	Sorafenib 400 mg BID	OS, ORR, AEs	August 27, 2021
		389/389	64/64	323(83.0)/ 337(86.6)		Durvalumab 1500 mg Q4W			
Li 2022 (14)	3	159/156	53/54	135(84.9)/ 141(90.4)	China (100.0)	FOLFOX-HAIC (oxaliplatin 130 mg/m2, leucovorin 400 mg/m2, fluorouracil bolus 400 mg/m2 on day 1, and fluorouracil infusion 2400 mg/m2 for 24 hours, Q3W)	TACE	OS, PFS, ORR, AEs	November 28, 2020
Llovet 2023 (36) (Leap-002)	3	395/399	66/66	317(80.3)/ 327(82.0)	Asia (30.7), Japan and Western (69.3)	Lenvatinib 8/12 mg QD + pembrolizumab 200 mg Q3W	Lenvatinib 8/12 mg QD + placebo	OS, PFS, ORR, AEs	June 21, 2022
Lu 2023 (37)	-	51/54	-	49(96.1)/ 48(88.9)	China (100.0)	Irradiation stent placement with 125I + TACE	Sorafenib 400 mg BID + TACE	OS, ORR, AEs	July 15, 2021
Lyu 2022 (38) (FOHAIC-1)	3	130/132	54/53	115(88.5)/ 123(93.2)	China (100.0)	HAIC-FO (oxaliplatin 130 mg/m2, leucovorin 200 mg/m2, fluorouracil 400 mg/m2, and fluorouracil 2400 mg/m2 Q3W)	Sorafenib 400 mg BID	OS, PFS, ORR, AEs	October 31, 2020
Meyer 2017 (39) (TACE 2)	3	157/156	65/68	139(88.5)/ 138(88.5)	United Kingdom (100.0)	DEB-TACE+Sorafenib 400 mg BID	DEB-TACE + placebo BID	OS, PFS, ORR	July, 2015
Park 2019 (40) (STAH)	3	170/169	60.2/61.3	136(80.0)/ 147(87.0)	South Korea (100.0)	Sorafenib 400 mg BID + TACE	Sorafenib 400 mg BID	OS, PFS, ORR, AEs	June, 2017
Peng 2023 (41) (LAUNCH)	3	170/168	54/56	139(81.8)/ 132(78.6)	China (100.0)	Lenvatinib 8/12 mg QD + TACE	Lenvatinib 8/12 mg QD	OS, PFS, ORR	October 10, 2021
Qin 2023 (10) (RATIONALE -301)	3	342/332	62/60	289(84.5)/ 281(84.6)	Asia (74.5), Others (25.5)	Tislelizumab 200 mg Q3W	Sorafenib 400 mg BID	OS, PFS, ORR, AEs	July 11, 2022
Qin 2025 (42) (CARES-310)	3	272/271	58/56	227(83.5)/ 230(84.9)	Asia (82.7), Others (17.3)	Camrelizumab 200 mg Q2W + rivoceranib 250 mg QD	Sorafenib 400 mg BID	OS, PFS, ORR, AEs	June 14, 2023
Ren 2021 (43) (ORIENT-32)	2/3	380/191	53/54	334(87.9)/ 171(89.5)	China (100.0)	Sintilimab 200 mg Q3W + IBI305–15 mg/kg Q3W	Sorafenib 400 mg BID	OS, PFS, ORR, AEs	August 15, 2020
Sangro 2025 (19) (EMERALD-1)	3	204/205	64.5/66	162(79.4)/ 163(79.5)	Asia (59.6), Others (40.4)	TACE + Durvalumab 1500 mg Q4W followed by durvalumab 1120 mg Q3W + bevacizumab 15 mg/kg Q3W	TACE + placebo Q4W followed by placebo Q3W	PFS, ORR, AEs	September 11, 2023
		207/205	65/66	156(75.4)/ 163(79.5)		TACE + Durvalumab 1500 mg Q4W followed by durvalumab 1120 mg Q3W + placebo Q3W			
Shi 2023 (44)	–	45/45	58.9/60.6	40(88.9)/ 39(86.7)	China (100.0)	DEB-TACE + CalliSpheres^®^ microspheres	TACE	OS, PFS	March,2022
Shi 2025 (45) (HEPATORCH)	3	162/164	58/56	134(82.7)/ 148(90.2)	China (96.3), Others (3.7)	Toripalimab 240 mg Q3W + bevacizumab 15 mg/kg Q3W	Sorafenib 400 mg BID	OS, PFS, ORR, AEs	May 31, 2024
Yau 2022 (11) (CheckMate459)	3	371/372	65/65	314(84.6)/ 317(85.2)	Asia (39.7), Others (60.3)	Nivolumab 240 mg Q2W	Sorafenib 400 mg BID	OS, PFS, ORR, AEs	April 17, 2019
Yau 2024 (46) (COSMIC-312)	3	432/217	64/64	360(83.3)/ 186(85.7)	Asia (28.8), Others (71.2)	Cabozantinib 40 mg QD + atezolizumab 1200 mg Q3W	Sorafenib 400 mg BID	OS, PFS, ORR, AEs	November 30, 2021
		188/ 217	64/64	158(84.0)/ 186(85.7)		Cabozantinib 60 mg QD			
Zhao 2025 (47)	2/3	230/116	57/56	197(85.7)/ 104(89.7)	China (100.0)	Finotonlimab 200 Q3W + SCT510–15 mg/kg Q3W	Sorafenib 400 mg BID	OS, PFS, ORR, AEs	November 02, 2023
Zheng 2022 (48)	2	32/32	56/52	30(93.8)/ 31(96.9)	China (100.0)	Sorafenib 400 mg BID + 3cir-OFF HAIC (oxaliplatin 35 mg/m2, 5-fluorouracil infusion 600 mg/m2 Q3W)	Sorafenib 400 mg BID	OS, PFS, ORR, AEs	December 28, 2020
Zhou 2024 (49)	–	82/81	55/55	74(90.2)/ 75(92.6)	China (100.0)	DEB-TACE	TACE	OS, PFS, ORR	July 28, 2022
Zhou 2025 (50) (APOLLO)	3	433/216	57/56	371(85.7)/ 180(83.3)	China (100.0)	Anlotinib 10 mg QD on days 1-14+ penpulimab 200 mg on day 1 Q3W	Sorafenib 400 mg BID	OS, PFS, ORR, AEs	January 29, 2024

**Table 2:** Baseline characteristics of studies included in the network meta-analysis (2).

Study	BCLC (A/B/C)	Child-Pugh (A/B/C)	ECOG (0/1/2)	Etiology (HBV/HCV/non-viral)	ALBI (1/2/3)	AFP ≥400ng/mL	EHS	PVTT	MVI	PVI
Chen 2025 (24)	-	-	-	-	-	-	-	25/11	-	-
	-	-	-	164/72/100	-					
Cheng 2022 (5) (IMbrave150)	0/51/277	–	209/127/0	–	–	126/61	212/93	–	129/71	–
	0/25/134	–	103/62/0	76/36/53	–					
Ding 2021 (25)	-	22/10/0	24/8/0	10959	12/20/0	16/18	13/9	32/32	-	-
	-	28/4/0	22/10/0	29/3/0	11/21/0					
Dong 2025 (18) (TALENTACE)	–	171/0/0		–	–	–	–	–	–	–
	–	171/0/0	–	–	–					
Duan 2024 (26)	0/48/74	104/18/0	76/46/0	99/4/19	-	53/43	17/18	-	-	64/70
	0/40/81	101/20/0	70/51/0	106/2/13	-					
Finn 2025 (27) (IMbrave152)	–	–	–	–	–	–	–	–	–	–
	–	–	–	–	–					
Finn 2025 (28) (MORPHEUS -Liver)	2/7/31	39/1/0	18/22/0	11/13/16	-	13/12	-	-	-	-
	0/6/12	18/0/0	13/5/0	4/8/6	-					
He 2019 (15) (SoraHAIC)	-	-	12/79/34	100/6/19	-	-	38/42	-	-	125/122
	-	-	9/83/30	99/7/16	-					
Ikeda 2016 (29)	0/19/46	57/8/0	50/15/0	22/18/NA	-	-	19/13	40/17	-	-
	0/16/25	39/2/0	33/8/0	9/20/NA	-					
Kondo Kondo 2019 (30) (SCOOP-2)	2/14/19	31/4/0	-	3/21/11	-	-	10/8	21/22	-	-
	2/13/18	29/4/0	-	4/20/10	-					
Kudo 2018 (31) (SILIUS)	0/32/70	90/12/0	89/13/0	26/47/NA	-	49/46	27/26	-	-	-
	0/27/76	93/10/0	91/12/0	22/46/NA	-					
Kudo 2018 (32) (REFLECT)	0/104/374	475/3/0	304/174/0	251/91/74	–	–	291/295	–	–	109/90
	0/92/374	471/5/0	301/175/0	228/126/53	–					
Kudo 2022 (33) (TACTICS)	27/44/9	79/1/0	-	10/38/32	-	-	-	-	-	-
	33/34/9	71/5/0	-	2/53/21	-					
Kudo 2024 (17) (LEAP-012)	80/135/21	237/0/0	216/21/0	42/153/54	171/65/0	37/40	–	–	–	–
	68/146/29	243/0/0	213/30/0	39/144/75	174/69/0					
Lai 2025 (34) (SHATA-001)	-	-	-	132/NA/NA	-	-	32/22	116/48	-	-
	-	-	-	64/NA/NA	-					
Lau 2025 (35) (HIMALAYA)	0/77/316	387/4/2	244/148/1	122/110/161	217/174/1	145/124/137	209/203/212	–	103/100/94	–
	0/66/323	379/10/0	241/147/1	119/104/166	203/185/1					
	0/80/309	380/8/1	237/150/2	119/107/163	198/189/2					
Li 2022 (14)	-	159/0/0	95/64/0	140/2/17	-	76/81	-	-	-	-
	-	156/0/0	102/54/0	141/3/12	-					
Llovet 2023 (36) (Leap-002)	0/85/310	393/1/0	267/127/0	192/94/146	–	119/132	249/243	–	71/62	–
	0/95/302	397/0/0	271/126/0	193/87/153	–					
Lu 2023 (37)	-	24/27/0	12/27/12	39/3/9	-	-	-	51/54	-	-
	-	26/28/0	17/23/14	47/2/5	-					
Lyu 2022 (38) (FOHAIC-1)	0/5/125	88/42/0	–	120/2/8	64/66/0	69/64	44/46	–	94/91	10/13
	0/9/123	93/39/0	–	114/4/14	71/61/0					
Meyer 2017 (39) (TACE 2)	-	145/5/0	98/58/0	7/15/89	-	-	-	-	-	-
	-	148/3/0	97/58/0	7/9/87	-					
Park 2019 (40) (STAH)	3/39/128	148/22/0	136/33/1	134/8/144	–	–	62/59	–	–	–
	0/44/125	147/22/0	140/28/1	120/16/149	–					
Peng 2023 (41) (LAUNCH)	-	-	89/81/0	148/4/18	41/129/0	83/87	94/95	-	-	122/117
	-	-	99/69/0	144/6/18	53/115/0					
Qin 2023 (10) (RATIONALE -301)	0/70/272	340/1/0	183/159/0	203/46/NA	256/81/1	135/116	219/198	–	51/49	–
	0/80/252	332/0/0	181/151/0	206/39/NA	226/98/0					
Qin 2025 (42) (CARES-310)	0/38/234	272/0/0	120/152/0	208/22/42	-	96/100	175/180	-	40/52	-
	0/40/231	271/0/0	116/155/0	197/29/45	-					
Ren 2021 (43) (ORIENT-32)	0/56/324	365/15/0	183/197/0	359/6/NA	–	165/81	279/144	–	105/50	–
	0/27/164	182/9/0	91/100/0	179/8/NA	–					
Sangro 2025 (19) (EMERALD-1)	51/117/35	200/4/0	167/37/0	75/42/86	-	-	-	-	16/13/13	-
	49/122/31	201/4/0	175/30/0	75/54/76	-					
	59/114/33	201/6/0	173/34/0	70/48/88	-					
Shi 2023 (44)	17/28/0	33/12/0	17/28/0	–	–	–	–	–	–	–
	14/31/0	29/16/0	13/32/0	–	–					
Shi 2025 (45) (HEPATORCH)	0/33/129	162/0/0	92/70/0	147/7/2	-	79/75	87/82	-	62/57	-
	0/37/127	164/0/0	93/71/0	146/8/5	-					
Yau 2022 (11) (CheckMate459)	15/53/303	365/NA/NA	271/99/0	116/87/168	–	124/142	222/207	–	–	–
	18/63/291	357/NA/NA	261/111/0	117/86/168	–					
Yau 2024 (46) (COSMIC-312)	0/140/292	432/0/0	276/154/1	127/136/169	249/182/1	163/65/65	232/123/103	-	136/61/67	-
	0/72/145	217/0/0	143/74/0	64/67/86	123/89/3					
	0/66/122	188/0/0	126/62/0	59/60/69	103/82/2					
Zhao 2025 (47)	0/46/184	214/16/0	105/125/0	208/10/NA	–	109/55	142/69	–	85/51	–
	0/23/93	108/8/0	53/63/0	100/7/NA	–					
Zheng 2022 (48)	-	28/4/0	14/16/2	28/2/NA	15/17/0	-	4/5	-	-	-
	-	27/5/0	14/15/3	29/3/NA	12/20/0					
Zhou 2024 (49)	–	71/11/0	36/46/0	74/NA/NA	40/37/NA	39/45	24/28	82/81	–	–
	–	66/15/0	31/50/0	67/NA/NA	33/44/NA					
Zhou 2025 (50) (APOLLO)	0/79/353	399/33/0	247/186/0	365/16/NA	267/166/0	-	267/137	-	179/87	-
	0/42/174	201/15/0	122/94/0	181/7/NA	142/74/0					

BCLC, Barcelona Clinic Liver Cancer; ECOG, Eastern Cooperative Oncology Group; HBV, Hepatitis B Virus; HCV, Hepatitis C Virus; ALBI, Albumin-bilirubin; AFP, Alpha-fetoprotein; EHS, Extrahepatic Spread; PVTT, Portal Vein Tumor Thrombus; MVI, Microvascular Invasion; PVI, Portal Vein Invasion.

**Table 1** summarizes the characteristics of the 35 studies incorporated into this analysis. The majority comprised Phase 3 RCTs, including key trials such as IMbrave150, HIMALAYA, and LEAP-002. In addition, several Phase Ib/II trials, such as TACTICS and SCOOP-2, were also incorporated. Notably, three studies (HIMALAYA, EMERALD-1, and COSMIC-312) featured a three-arm design. Sample sizes exhibited substantial variability, ranging from small exploratory studies with fewer than 50 participants (e.g., Chen 24) to large-scale, multi-center clinical trials exceeding 1,000 patients (e.g., HIMALAYA). The 35 studies collectively comprised 13,595 patients, with a median age of 61.2 years. Males accounted for 11,264 (82.9%) of the participants, with male proportions exceeding 90% in some studies (e.g., Lu 2023 (37), Zheng 2022 (48)). This aligns with the known epidemiological characteristics of the broader hepatocellular carcinoma patient population. Geographically, Asian countries were the predominant contributors to the research landscape, with a substantial number of studies conducted exclusively in China or Japan. Other studies were global multi-center trials, recruiting populations from Asia, Europe, North America, and other regions. Notably, the geographical distribution revealed a distinct age stratification trend, with Chinese patient cohorts exhibiting a significantly younger age of onset compared to patients in Japanese and global (predominantly European and North American) multi-center studies. The enrolled patients received one of 30 distinct treatment regimens: Tiragolumab+Atezolizumab+Bevacizumab (Tira-Atezo-Beva); TACE+Atezolizumab+Bevacizumab (TACE-Atezo-Beva); Radiotherapy+Toripalimab (RT-Tori); Durvalumab+Tremelimumab (Durva-Trem); Camrelizumab+Rivoceranib (Camre-Rivo); Anlotinib+Penpulimab (Anlo-Penpu); Finotonlimab+SCT510.

(Fino-SCT510); Toripalimab+Bevacizumab (Tori-Beva); Atezolizumab+Cabozantinib (Atezo-Cabo); Tislelizumab (Tisle); Sintilimab+IBI305 (Sinti-IBI305); Pembrolizumab+Lenvatinib (Pembro-Lenv); Atezolizumab+Bevacizumab (Atezo-Beva); Nivolumab (Nivo); TACE+Durvalumab+Bevacizumab (TACE-Durva-Beva); Lenvatinib+Pembrolizumab+TACE (Lenv-Pembro-TACE); TACE+Lenvatinib (TACE-Lenv); HAIC (HAIC); HAIC+Sorafenib (HAIC-Sora); ISP+TACE (ISP-TACE); DEB-TACE (DEB-TACE); DEB-TACE+Apatinib (DEB-TACE-Apa); Lenvatinib (Lenv); TACE+Sorafenib (TACE-Sora); DEB-TACE+Sorafenib (DEB-TACE-Sora); TACE (TACE); Sorafenib (Sora); Durvalumab (Durva); TACE+Durvalumab (TACE-Durva); and Cabozantinib (Cabo). Regarding outcome measures, the majority of studies reported OS, PFS, and ORR, thus providing robust data for the network meta-analysis.

**Table 2** details the clinicopathological characteristics of the included studies. Across the included studies, patients generally exhibited favorable baseline liver function. Most studies primarily enrolled Child-Pugh A class patients, with only a limited proportion of Child-Pugh B class patients included in a few studies (e.g., SoraHAIC, Lu 2023 (37), Duan 2024 (26)). Some studies (e.g., HIMALAYA, LEAP-012, APOLLO) also reported ALBI grading, with participants predominantly categorized as ALBI grade 1 or 2. Regarding performance status (ECOG PS), participants were primarily ECOG 0 or 1, and ECOG 2 patients were generally excluded from most Phase III clinical trials. Disease staging was closely associated with the study design: in studies evaluating immunotherapy, patients were predominantly BCLC stage C, frequently presenting with a high proportion of extrahepatic spread (EHS) and/or macrovascular invasion (MVI). Conversely, in studies assessing TACE or adjuvant therapies (e.g., EMERALD-1, TACE 2), BCLC stage B patients comprised a larger proportion. Notably, the etiological background of HCC demonstrated significant heterogeneity across studies, consistent with the geographical distribution patterns outlined in **Table 1**. In studies predominantly conducted in China (e.g., ORIENT-32, RATIONALE-301, CARES-310, APOLLO), hepatitis B virus (HBV) infection was the predominant primary etiology (typically >80%). In contrast, in Western-led or global multi-center trials (e.g., TACE 2, HIMALAYA, CheckMate459), the proportion of hepatitis C virus (HCV) infection and non-viral etiologies (such as alcoholic or metabolic-associated liver disease) was substantially higher. Furthermore, some studies meticulously documented specific details concerning alpha-fetoprotein (AFP) levels, portal vein tumor thrombosis (PVTT), and portal vein invasion—all indicators reflecting tumor burden and its invasive characteristics.

### 3.2 Quality assessment of included studies

The methodological quality of the 35 included RCTs was rigorously assessed using the Cochrane Risk of Bias Tool (RoB 2.0). Overall, the methodological quality of these studies was deemed high (**Supplementary Figures 1**, **2**). Specifically, regarding “Overall bias,” 29 studies were categorized as “Low risk,” while 6 were assigned “Some concerns,” and no studies were classified as “High risk.” All included studies consistently demonstrated “Low risk” in the domains of “Missing outcome data,” “Measurement of the outcome,” and “Selection of the reported result.” The primary sources of bias risk were predominantly concentrated within the “Randomization process” and “Deviations from intended interventions” domains.

In the “Randomization process” domain, two studies, Chen 2025 (24) and Dong 2025 (18), were reported as conference abstracts. Although randomization was mentioned, explicit details regarding random sequence generation and allocation concealment mechanisms were not provided, prompting an assessment of “Some concerns.” Similarly, despite MORPHEUS-Liver employing block randomization, the authors themselves acknowledged “Baseline imbalances” within the text, which also led to a “Some concerns” rating for this domain.

The “Some concerns” identified in the “Deviations from intended interventions” domain largely stemmed from the open-label nature of these studies. Blinding of participants and personnel was frequently impractical due to fundamental differences in the administration of treatment regimens. For instance, Dong 2025 (18) and Kudo 2018 (SILIUS) (31) augmented systemic therapies with HAIC, which necessitates arterial cannulation; Chen 2025 (24) and Li 2022 (14) compared drug treatment arms with those involving invasive TACE procedures; and Lai 2025 (34) and Finn 2025 (IMbrave152) (27) and Finn 2025 (MORPHEUS-Liver) (28) supplemented standard treatment with additional intravenous infusions of Tiragolumab. The inherent asymmetry in drug delivery routes and treatment modalities rendered blinding unfeasible in these specific contexts. Despite these isolated concerns, the overall credibility of this meta-analysis remains high, given that the vast majority of studies were rated as “Low risk” and none were designated “High risk”.

### 3.3 Network meta-analyses

#### 3.3.1 Comparisons of OS and PFS

**Figure 2:**
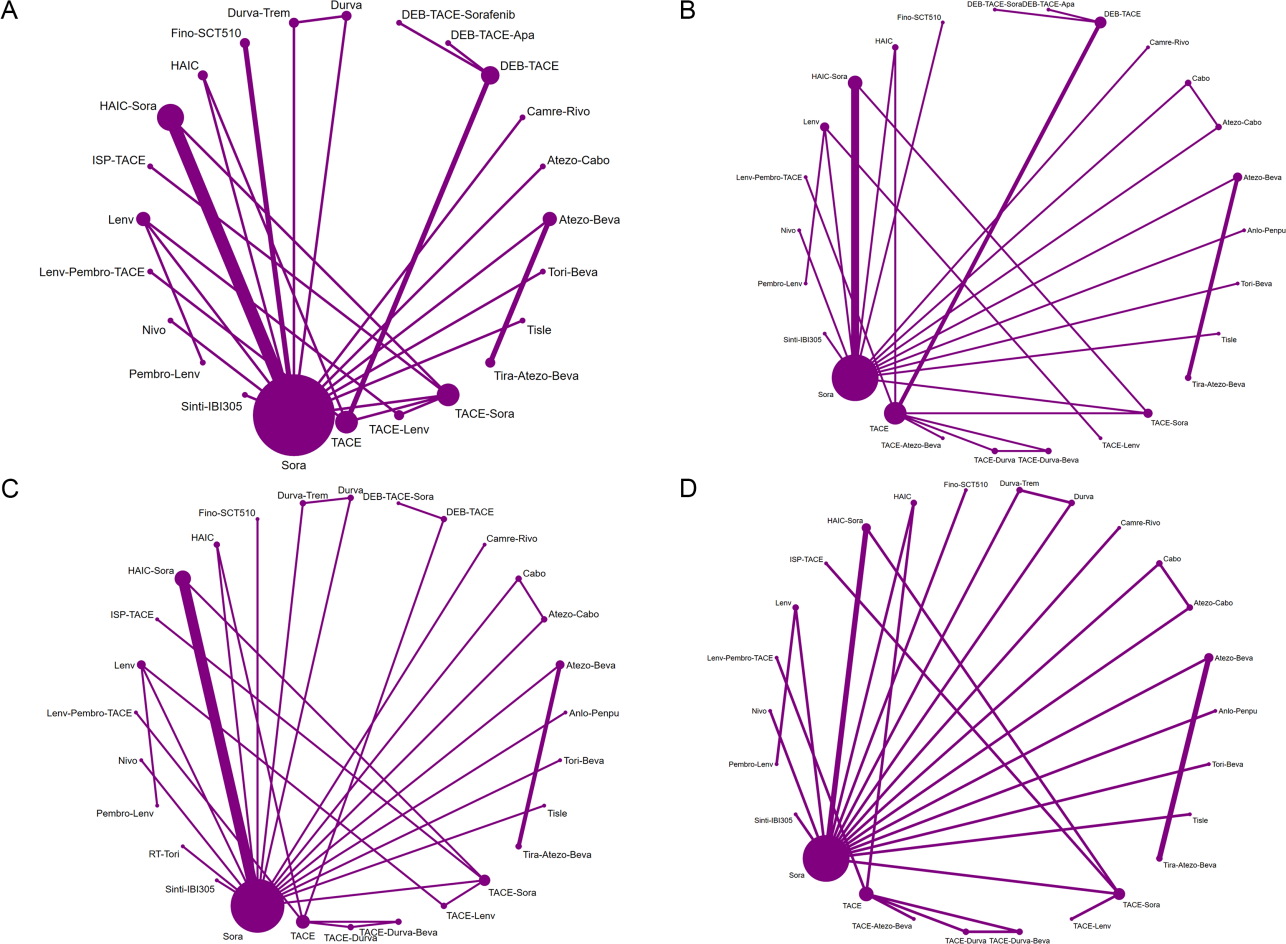
Network plots of treatment comparisons for overall survival **(A)**, progression-free survival **(B)**, objective response rate **(C)**, and grade ≥3 AEs **(D)**.

**Figure 3:**
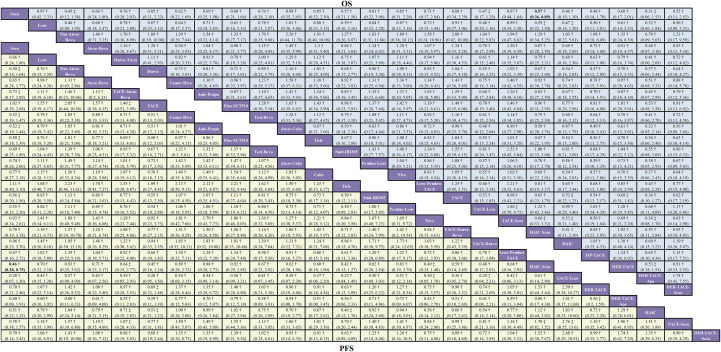
Network meta-analysis league table for the comparative efficacy of treatments on overall survival (OS) and progression-free survival (PFS) with GRADE evidence certainty. Pooled HR (95% credible interval) for OS (upper triangle) and PFS (lower triangle). Each cell contained an HR (95% credible intervals) comparing the row-defining treatment to the column-defining treatment. Comparisons are shown from left to right. For PFS, an HR of < 1 favors column-defining treatment. For OS, an HR of less than one favors row-defining treatment. The significant results are shown in bold.*=high certainty of evidence; †=moderate certainty of evidence; ‡=low certainty of evidence; §=very low certainty of evidence.

For OS, 32 studies comprising 12,413 participants and 25 distinct treatment regimens were included (**Figure 2A**). The league table (**Figure 3**) indicated that only HAIC-Sora (HR = 0.57, 95% CrI: 0.36–0.89) demonstrated a statistically significant survival benefit compared to Sora monotherapy, a finding supported by moderate-quality evidence. While other regimens, including Atezo-Beva (HR = 0.66, 95% CrI: 0.24–1.80), HAIC (HR = 0.46, 95% CrI: 0.19–1.10), and TACE-Lenv (HR = 0.47, 95% CrI: 0.17–1.29), showed numerical improvements in OS compared to Sora, these differences did not achieve statistical significance. A direct comparison between Lenv and Sora monotherapies (HR = 0.97, 95% CrI: 0.42–2.31) revealed no statistically significant difference, a finding underpinned by moderate-quality evidence. Based on SUCRA values (**Supplementary Table 3**) and Bayesian ranking probabilities (**Supplementary Figure 11**) for OS, the top four ranked treatments were DEB-TACE-Apa (82.7%), HAIC (73.5%), TACE-Lenv (71.7%), and Tira-Atezo-Beva (71.7%).

For PFS, 30 studies, including 12,151 participants and 26 treatment regimens, were analyzed (**Figure 2B**). Analysis of PFS revealed that several combination regimens demonstrated significantly superior efficacy compared to Sora monotherapy. HAIC-Sora significantly reduced the risk of disease progression (HR = 0.46, 95% CrI: 0.28–0.75), a statistically significant finding supported by moderate-quality evidence (**Figure 3**). TACE-Lenv demonstrated a substantial PFS benefit (HR = 0.28, 95% CrI: 0.07–1.20), suggesting notable efficacy, although the broad credible interval precluded statistical significance. Similar to OS, the PFS comparison between Lenv and Sora (HR = 0.66, 95% CrI: 0.24–1.80) did not show a statistically significant difference. While the HR for Tira-Atezo-Beva compared to Sora (HR = 0.50, 95% CrI: 0.14–1.64) was numerically favorable, the wide credible interval and low-quality evidence grading indicated considerable uncertainty in this result. According to SUCRA ranking (**Supplementary Table 3**) and Bayesian ranking probabilities (**Supplementary Figure 12**), TACE-Lenv ranked first for PFS (85.5%), followed by DEB-TACE-Apa (74.1%), HAIC-Sora (73.2%), and HAIC (65.7%).

The combined findings from the network meta-analyses for OS and PFS consistently highlighted that HAIC-Sora was the sole combination regimen demonstrating statistically significant superiority over Sora monotherapy for both endpoints, supported by moderate-quality evidence. Conversely, other combination regimens (e.g., Atezo-Beva, TACE-Lenv), despite exhibiting numerical trends towards risk reduction (HRs ranging from 0.3 to 0.7), did not achieve statistical significance due to wide CrIs.

#### 3.3.2 Comparisons of ORR and grade ≥3 AEs

**Figure 4:**
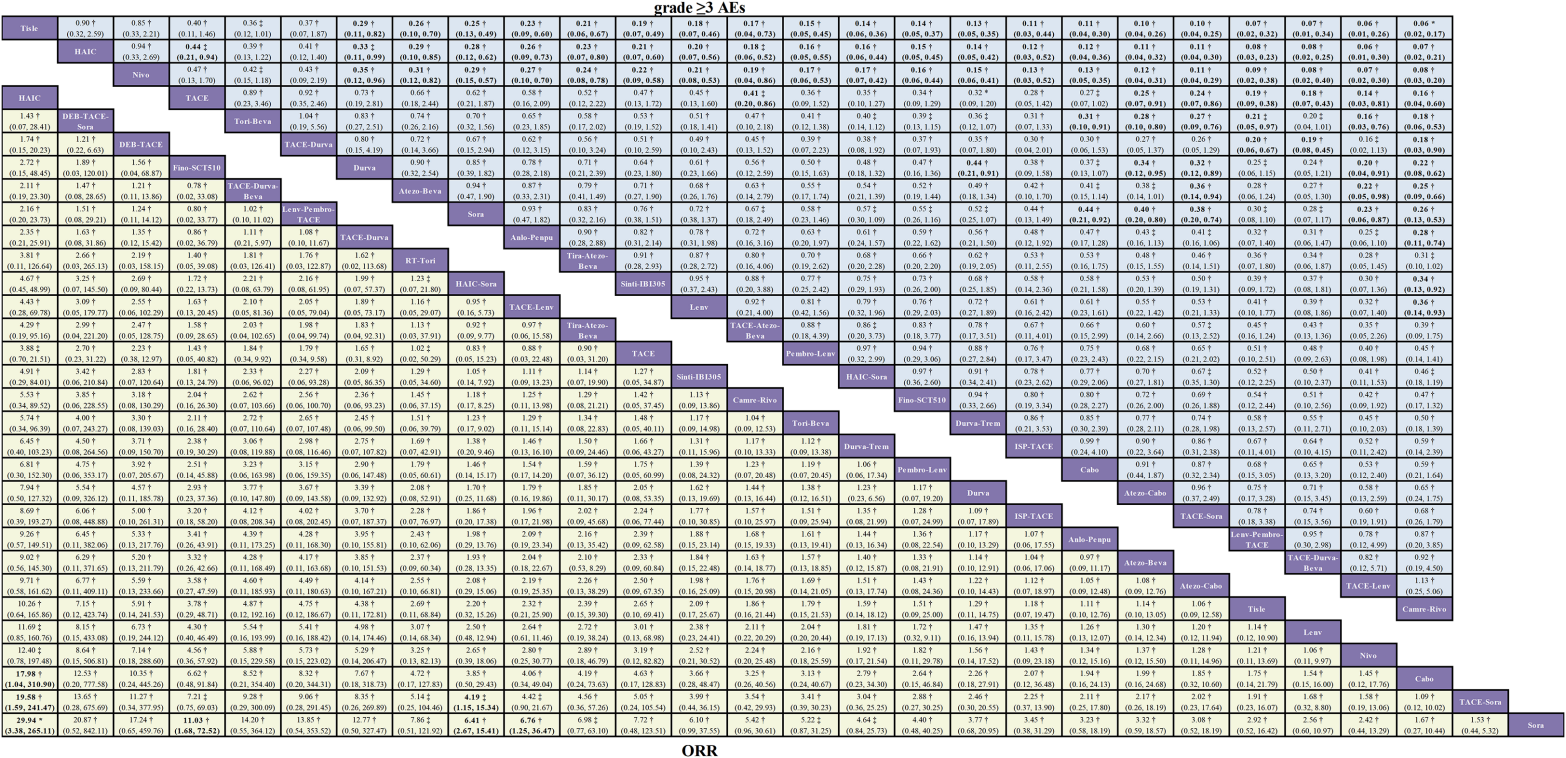
Network meta-analysis league table for the comparative efficacy of treatments on objective response rate (ORR) and grade ≥3 adverse events (AEs) with GRADE evidence certainty. Pooled OR (95% credible interval) for grade ≥3 AEs (upper triangle) and ORR (lower triangle). Each cell contained an OR (95% credible intervals) comparing the row-defining treatment to the column-defining treatment. Comparisons are shown from left to right. For grade ≥3 AEs, an OR of < 1 favors row-defining treatment. are shown from left to right. For ORR, an OR of > 1 favors column-defining treatment. The significant results are shown in bold. *=high certainty of evidence; †=moderate certainty of evidence; ‡=low certainty of evidence; §=very low certainty of evidence.

For ORR, 31 studies comprising 28 distinct treatment regimens were included (**Figure 2C**). The league table (**Figure 4**) revealed that HAIC demonstrated the most substantial improvement in ORR compared to Sora monotherapy (OR = 29.94, 95% CrI: 3.38–265.11). However, its wide credible interval suggests limited precision in the effect size estimate, despite its high-quality evidence grading. This imprecision, while not negating the observed trend, underscores the instability of the estimate and the need for more robust data. Additionally, Fino-SCT510 (OR = 11.03, 95% CrI: 1.68–72.52), HAIC-Sora (OR = 6.41, 95% CrI: 2.67–15.41), and TACE-Lenv (OR = 6.76, 95% CrI: 1.25–36.47) also exhibited significant ORR improvements versus Sora. Furthermore, HAIC demonstrated statistically significant ORR improvements when compared individually with Cabo (OR = 17.98, 95% CrI: 1.04–3310.90) and TACE-Sora (OR = 19.58, 95% CrI: 1.59–241.47). TACE-Sora also yielded a statistically significant ORR benefit versus HAIC-Sora (OR = 4.19, 95% CrI: 1.15–15.34), though its lower evidence grading translated to reduced credibility. Based on SUCRA values (**Supplementary Table 3**) and Bayesian ranking probabilities (**Supplementary Figure 13**), the top four ranked treatments for ORR were HAIC (84.2%), DEB-TACE-Sora (72.6%), DEB-TACE (71.2%), and TACE-Durva-Beva (68.0%).

For grade ≥3 AEs, 25 studies comprising 27 treatment regimens were included (**Figure 2D**). Detailed analysis of the league table (**Figure 4**) revealed that several regimens demonstrated a more favorable profile for the incidence of grade ≥3 AEs compared to Sora monotherapy. Among these, TACE-Lenv exhibited the most substantial reduction in the occurrence of grade ≥3 AEs (OR = 0.23, 95% CrI: 0.06–0.87). This statistically significant result was supported by moderate GRADE evidence, indicating high credibility. Other regimens, such as Camre-Rivo (OR = 0.26, 95% CrI: 0.13–0.53) and Tisle (OR = 0.25, 95% CrI: 0.13–0.49), also demonstrated statistically significant reductions in the incidence of grade ≥3 AEs compared to Sora, with high-quality evidence. According to SUCRA ranking (**Supplementary Table 3**) and Bayesian ranking probabilities (**Supplementary Figure 14**), the Tisle regimen ranked first for the lowest incidence of grade ≥3 AEs (91.3%), followed by HAIC (90.8%), Nivolumab (Nivo) (89.0%), and TACE (74.0%).

The combined results from the network meta-analysis for ORR and grade ≥3 AEs indicate that HAIC demonstrated superior ORR and a favorable safety profile, positioning it as a therapeutic strategy with a strong overall balance of efficacy and safety.

#### 3.3.3 Heterogeneity and inconsistency

Model convergence was rigorously confirmed via diagnostic plots and Gelman-Rubin diagnostic statistics (**Supplementary Figures 3**-**S10**), all indicating stable and reproducible MCMC inference across chains. As presented in **Supplementary Table 4**, the differences in DIC values between the fixed-effects and random-effects models were consistently less than 5 across all outcome measures. This finding indicated comparable goodness-of-fit for both models, underscoring the robustness and comparability of our results.

To evaluate the consistency assumption of the network, DIC values were further compared between consistency and inconsistency models. As shown in **Supplementary Table 5**, the differences in DIC values between the consistency and inconsistency models for all outcomes were consistently below 5. This observation suggests a lack of significant global inconsistency within the network, implying good agreement between direct and indirect evidence. Furthermore, the node-splitting method further confirmed no significant local inconsistency, with all node-splitting P-values exceeding 0.05. Additionally, low I² values across all outcomes indicated minimal statistical heterogeneity among the included studies, thereby supporting the overall homogeneity and stability of the network.

Potential publication bias was evaluated using comparison-adjusted funnel plots for all outcome measures. These plots indicated no obvious asymmetry **(Supplementary Figure 15**-**S18**). However, given the limited number of comparisons, conclusions derived from this assessment should be interpreted with caution.

#### 3.3.4 Network GRADE classification

The assessment of evidence quality, conducted using the CINeMA framework (**Figure 3**), revealed variations in its distribution across different outcome measures. For OS, among 300 comparisons, the vast majority (288) were classified as “Moderate.” Only 10 and 2 comparisons received “Low” and “Very Low” ratings, respectively. PFS assessments followed a similar pattern: out of 325 comparisons, 306 were deemed “Moderate,” while 18 and 1 were categorized as “Low” and “Very Low,” respectively.

Notably, “High” quality evidence was observed for ORR and Grade ≥3 AEs. Specifically, among the 378 ORR comparisons, one was rated “High,” 364 “Moderate,” and 13 “Low.” Similarly, for the 325 comparisons involving Grade ≥3 AEs, two received a “High” rating, 292 were “Moderate,” and 31 “Low.”.

## 4 Discussion

### 4.1 Main findings

This network meta-analysis comprehensively synthesized direct and indirect evidence to compare the efficacy and safety of monotherapies and combination regimens, including ICIs and interventional therapies, for the first-line treatment of uHCC. Encompassing 35 RCTs with 13,595 patients and 30 distinct treatment regimens, this study represents one of the largest and most comprehensive network meta-analyses in this specific field to date. We systematically evaluated four key outcome measures—OS, PFS, ORR, and Grade ≥3 AEs—across these regimens. The analysis involved 32 studies for OS, 30 for PFS, 31 for ORR, and 25 for Grade ≥3 AEs, incorporating diverse therapeutic approaches such as ICIs (e.g., Camrelizumab, Pembrolizumab), anti-VEGF agents (e.g., Sorafenib, Lenvatinib), and interventional therapies like HAIC and TACE.

Our primary findings indicate that HAIC-Sora consistently demonstrated the most significant and robust benefits in both OS and PFS compared to Sora monotherapy, supported by moderate-quality evidence. Notably, this was the sole combination regimen showing statistically significant superiority for survival endpoints over sorafenib. While SUCRA rankings further highlighted DEB-TACE-Apa, HAIC, and TACE-Lenv as consistently among the top-ranked treatments for both OS and PFS, it is important to note that these rankings are derived from a network meta-analysis that includes indirect evidence, and these specific comparisons did not achieve statistical significance in pairwise analyses against sorafenib. Regarding ORR, HAIC monotherapy exhibited a highly significant efficacy, notably superior to Sora and other regimens, and was underpinned by high-quality evidence. Moreover, Finotonlimab+SCT510, HAIC-Sora, and TACE-Lenv also yielded convincing improvements in ORR. Concerning safety, moderate-certainty evidence indicated that several regimens (e.g., Camre-Rivo, Tislelizumab) presented a significantly lower incidence of Grade ≥3 AEs compared to Sora, with TACE-Lenv demonstrating the lowest risk among all treatments. Collectively, HAIC-Sora emerged as a particularly promising regimen, excelling in both survival benefits and a favorable safety profile, thereby positioning it as an optimal strategy balancing efficacy and safety. For other promising regimens, further direct and statistically significant evidence is needed to confirm their superiority.

### 4.2 Analysis of results

The distinct survival benefits observed in this study can be elucidated through several biological and clinical mechanistic perspectives. The notable efficacy of the HAIC-Sora regimen likely arises from the synergistic interplay between local and systemic therapeutic modalities (51). HAIC, through direct intra-arterial perfusion of chemotherapeutic agents into the hepatic circulation, achieves local drug concentrations in the tumor microenvironment manifold higher than those attainable with systemic intravenous chemotherapy. This sustained, high-concentration exposure enhances direct cytotoxic effects on tumor cells (52). Concurrently, the hypoxic-ischemic microenvironment generated by HAIC may potentiate Sorafenib’s anti-angiogenic properties, fostering synergistic anti-tumor activity (53). Moreover, Sorafenib, through its inhibition of VEGFR and RAF kinase pathways, not only curtails tumor angiogenesis but also possesses immunomodulatory capabilities, potentially synergizing with HAIC-mediated immunogenic cell death (ICD) (54). Nevertheless, these mechanistic hypotheses warrant further validation through dedicated preclinical and translational studies, including comprehensive biomarker analyses.

The rationale for combining ICIs with interventional therapies hinges on the premise that interventional procedures can induce tumor antigen release, thereby augmenting the anti-tumor immune response (55). Indeed, prior research (56) suggests that TACE functions as a regional ICD inducer in HCC. The combined effects of chemotherapy and ischemic damage lead to the release of damage-associated molecular patterns (DAMPs) and pro-inflammatory cytokines, which in turn activate adaptive immunity and correlate with improved prognosis (57). TACE has been shown to reduce the density of intratumoral exhausted (CD8+/PD-1+) and regulatory (CD4+/FOXP3+) T cells, thereby mitigating immunosuppression and associating with improved recurrence-free survival (58). Transcriptomic analyses further reveal an upregulation of pro-inflammatory pathways (e.g., IRF2, interferon-related genes), fostering an inflammatory tumor microenvironment (TME) without necessarily affecting the expression of inhibitory molecules such as PD-L1 or IDO-1 (59). However, despite these compelling biological mechanisms, in the present study, TACE combined with ICI regimens (e.g., TACE-Atezo-Beva, TACE-Durva-Beva) did not achieve statistically significant survival benefits, even though numerical trends were observed. Several factors may account for this lack of statistical significance: firstly, the immune activation window post-TACE is often transient and subject to substantial inter-individual variability; secondly, the post-embolization hypoxic environment may paradoxically enhance the recruitment of immunosuppressive cells; and thirdly, the follow-up duration and/or sample sizes of the included studies might have been insufficient to definitively capture the long-term synergistic benefits of these combination therapies (60).

Regarding safety, ICI monotherapies (e.g., Tislelizumab, Nivolumab) exhibited a lower incidence of Grade ≥3 AEs compared to Sorafenib. This observation aligns with the fundamentally distinct mechanisms of action between ICIs, which modulate the immune system, and traditional cytotoxic or multi-kinase targeted drugs like Sorafenib. While ICIs primarily exert their therapeutic effects by reactivating the immune system, leading to immune-related adverse events (irAEs), the overall incidence of severe irAEs remains relatively low (61). In contrast, Sorafenib, a multi-target tyrosine kinase inhibitor, is frequently associated with a higher incidence of characteristic AEs, including hand-foot skin reaction, diarrhea, and hypertension (62). Remarkably, the TACE-Lenv regimen was associated with a significantly lower incidence of grade ≥3 AEs compared to Sora. While this finding might initially appear counterintuitive, several interwoven biological and clinical factors offer plausible explanations for this observed favorable safety profile.Firstly, Lenv and Sora possess distinct toxicity profiles. Data from the REFLECT trial (32), for instance, showed Sora to be associated with significantly higher rates of grade ≥3 hand-foot skin reaction. In contrast, Lenv, while linked to higher rates of hypertension, exhibited either comparable or lower rates for certain severe dermatologic and gastrointestinal toxicities when adjusted for exposure time. Secondly, TACE, as a locoregional therapy, primarily involves catheter-related complications rather than systemic toxicities. Therefore, its combination with Lenv does not necessarily contribute to the systemic AE burden in a straightforward additive fashion (63).Thirdly, concurrent TACE often mandates proactive dose reductions or interruptions of systemic Lenv to manage locoregional effects. This strategy consequently reduces cumulative systemic drug exposure and mitigates severe adverse events. Clinical experience further supports this approach, demonstrating that the combination can achieve effective tumor control with manageable toxicity through judicious dose adjustments and proactive AE management (64). Fourthly, emerging evidence suggests that Lenv promotes vascular normalization and modulates the tumor microenvironment, potentially reducing the incidence of certain ischemic-related complications often associated with TACE alone (65). Finally, a recent meta-analysis by Lei et al. (66) lends further support to this nuanced perspective. It demonstrated that while adding PD-1 inhibitors to TACE-Lenv significantly elevates the risk of grade ≥3 AEs, the TACE-Lenv dual combination itself maintains a more manageable safety profile, devoid of such incremental toxicity. It is crucial to underscore that this observation does not imply TACE-Lenv is without toxicity. Rather, it highlights that the toxicity profile of this combination differs qualitatively from that of Sora, thereby emphasizing the paramount importance of regimen-specific safety considerations in treatment selection.

### 4.3 Implications for clinical practice and research

Our findings provide significant clinical implications for the first-line treatment strategies of uHCC. This study highlights the therapeutic potential of both monotherapies and combination regimens, particularly those incorporating ICIs or interventional therapies, as first-line options for uHCC patients, especially those with BCLC stage C disease, Child-Pugh class A liver function, and ECOG performance status 0-1. Based on moderate-quality evidence, the HAIC-Sora combination regimen emerges as a strong candidate for consideration as a preferred option, particularly for patients presenting with portal vein invasion or portal vein tumor thrombosis, and in Asian populations where HAIC is more commonly practiced. This recommendation aligns with current liver cancer treatment guidelines in several Asian countries (including China, Japan, and South Korea) but has not yet been fully integrated into Western guidelines. The high-quality network meta-analysis evidence presented in this study is expected to significantly contribute to promoting the global clinical application of HAIC and facilitating future guideline updates.

For patients unsuitable for or unable to receive interventional therapies, established immunotherapy-targeted therapy combinations (e.g., Atezo-Beva, Camre-Rivo, Sinti-IBI305) continue to be pivotal first-line choices. While our network meta-analysis did not demonstrate statistically significant survival benefits for these regimens over Sora monotherapy, their established efficacy from landmark RCTs (e.g., IMbrave150, ORIENT-32, CARES-310) remains the basis for their widespread acceptance. Our network meta-analysis suggests the presence of subtle efficacy differences among various ICI-based combination regimens; however, current evidence remains insufficient for definitive conclusions regarding their superiority or inferiority relative to each other. Consequently, personalized treatment selection necessitates a comprehensive evaluation of the patient’s etiological background (e.g., HBV vs. HCV vs. non-viral), tumor burden, prior treatment history, economic accessibility, and individual preferences.

Several key directions for future clinical research emerge from these findings. Firstly, there is an urgent need for more head-to-head RCTs to directly compare the efficacy and safety of different combination regimens, with a particular focus on their differential effectiveness within specific patient subgroups (e.g., etiological, tumor burden, and liver function subgroups). Secondly, it is essential to accrue longer-term follow-up data and real-world evidence to thoroughly assess the sustained benefits and long-term toxicities associated with these combination therapies. Thirdly, a deeper exploration of effect modifiers, such as baseline disease severity, optimal intervention dose and timing, and predictive biomarkers (e.g., PD-L1 expression, tumor mutational burden, AFP levels), is crucial to refine patient stratification and achieve truly individualized therapy. Finally, reinforcing adherence to trial reporting guidelines (e.g., CONSORT) is paramount to ensure comprehensive study registration, data transparency, and sharing, thereby bolstering evidence quality and expediting clinical translation.

### 4.4 Comparison with previous studies

This study distinguishes itself as one of the most prominent network meta-analyses to date, both in its research scale and methodological rigor, for evaluating first-line treatment regimens in uHCC. While a recent meta-analysis by Liu et al. (67) investigated transarterial intervention therapy combined with systemic therapy for uHCC, our study distinguishes itself in several crucial respects. Firstly, a fundamental methodological distinction sets our work apart. Liu ets al.’s study employed conventional systematic review and meta-analysis, an approach inherently limited to direct comparisons between a restricted number of treatment options. In stark contrast, our research leveraged network meta-analysis. The primary strength of network meta-analysis lies in its capacity to indirectly compare and rank multiple treatment regimens, even in the absence of direct head-to-head trials, via common comparators or bridging interventions. This methodological prowess enabled us to compare all 30 first-line interventions within a unified framework and assess their relative efficacies. For instance, we could compare the comparative efficacy of Atezo-Beva versus Atezo-Cabo, even in the absence of direct RCT data for these specific regimens. Secondly, our study presents a substantially broader and more comprehensive scope. Liu et al.’s meta-analysis was primarily constrained to evaluating the efficacy of TACE or HAIC combined with TKIs and/or ICIs, compared to TACE/HAIC monotherapy or TKI/ICI monotherapy for uHCC. Conversely, our study undertook an assessment of 30 distinct first-line interventions. Beyond TACE, HAIC, TKIs, and ICIs, our scope encompassed additional treatment modalities, including radiotherapy and Irradiation stent placement with ^125^I. Rather than a simplistic comparison of ‘combination’ versus ‘monotherapy,’ our analysis provided comprehensive head-to-head comparisons of specific TACE, HAIC, TKI, ICI regimens, other therapeutic modalities, and their diverse combinations. Furthermore, while Liu et al.’s study included 20 investigations, comprising 5,485 patients, our meta-analysis integrated 35 RCTs, enrolling 13,595 patients. This larger number of RCTs and nearly threefold greater patient sample size underpinned our analysis with a more powerful and robust statistical foundation. This increased statistical power mitigated the risk of spurious findings and consequently bolstered the reliability of our conclusions. Finally, our study provides a distinct advantage through its refined comparison of specific treatment regimens. Liu et al.’s conclusions remained generalized, primarily indicating that transarterial intervention therapy combined with systemic therapy significantly prolonged OS, PFS, and time to progression (TTP), while also improving ORR and disease control rate (DCR). Our study, conversely, meticulously detailed the head-to-head comparative results for all 30 distinct treatment regimens across OS, PFS, ORR, and ≥3 AEs. This granular quantification of specific combination therapies and their effects therefore provides clinicians with far more precise and actionable guidance for selecting optimal treatment strategies.

We also acknowledge a recent network meta-analysis by Su et al. (68) that focused on interventional therapies combined with immunotherapy, our study presents crucial distinctions. Firstly, the majority of evidence in Su et al.’s research originated from retrospective cohorts and single-arm trials, with only two RCTs included. This methodology inherently introduces selection bias and diminishes the certainty of evidence. In stark contrast, our study rigorously adhered to the inclusion of only RCTs, thereby ensuring that our conclusions are founded on the highest level of clinical evidence. Secondly, Su et al.’s work primarily highlighted the efficacy of TACE combined with targeted immunotherapy. Conversely, our analysis, by integrating the most up-to-date RCT data, uniquely revealed the superior performance of HAIC-based combination regimens (particularly HAIC-Sora) in improving OS. This exceptional efficacy has often been underestimated in previous studies, which were frequently confounded by the inclusion of a substantial volume of retrospective data.

Furthermore, prior network meta-analyses addressing advanced HCC treatment predominantly focused on comparing single categories of immunotherapy or targeted therapy, typically encompassing a more restricted number of studies and intervention nodes. For instance, the meta-analysis published by Tian et al. (69) in 2024 included only 11 studies with 255 patients, primarily comparing different immune combination regimens but notably omitting interventional therapies. Similarly, Wang et al.’s (64) 2024 study focused specifically on TACE combined with Lenvatinib and PD-1 inhibitors, without incorporating HAIC or other ICIs. By integrating a substantially larger dataset of 35 RCTs and 30 distinct treatment nodes, our study, for the first time, provides a panoramic and comprehensive comparison across immunotherapy, interventional therapy, and their various combination regimens.

Crucially, in the interpretation of results, this study underscores that treatment regimen recommendations should not be based solely on SUCRA rankings. Instead, a holistic consideration encompassing effect sizes, CrIs, and the certainty of evidence (e.g., GRADE/CINeMA assessment) is imperative. This perspective fully aligns with recent advancements in network meta-analysis methodology and best practices. This is particularly pertinent in our study, where only the comparison between HAIC-Sora and Sora monotherapy for survival outcomes achieved statistical significance. For example, while DEB-TACE-Apa achieved the highest SUCRA ranking for OS (82.7%) in our analysis, its direct comparison with Sora exhibited a wide credible interval and moderate-quality evidence and, importantly, did not reach statistical significance. Consequently, it cannot be simply designated as the “best regimen” without further qualification. In contrast, HAIC-Sora, despite not holding the top SUCRA ranking for some outcomes, demonstrated statistically significant efficacy supported by acceptable evidence quality, thus warranting a more robust clinical recommendation.

### 4.5 Strengths and limitations

This study rigorously adhered to stringent methodological standards, contributing significantly to its robustness. The study protocol was prospectively registered in PROSPERO, ensuring both transparency and reproducibility. Our comprehensive literature search strategy spanned four major databases—PubMed, EMBASE, Cochrane Library, and Web of Science—without language restrictions, thereby effectively minimizing potential search bias. The risk of bias for included studies, assessed using the Cochrane RoB 2.0 tool, indicated a generally high overall evidence quality: 29 studies were categorized as low risk, 6 as ‘some concerns,’ and none at high risk. The network meta-analysis employed a Bayesian framework and fixed-effects model, with parameter estimation performed using the MCMC method, consistently demonstrating good model convergence and stability. Crucially, consistency checks revealed no significant inconsistency within the network (DIC differences between consistency and inconsistency models were < 5 for all outcomes), signifying robust exchangeability between indirect and direct evidence. Heterogeneity assessment further confirmed low I² values across all outcomes, suggesting good homogeneity among the included studies. Publication bias, evaluated through funnel plot analysis, indicated no apparent asymmetry, suggesting a minimal impact on our findings. Finally, the CINeMA framework, a widely recommended tool for assessing the certainty of evidence in network meta-analysis, was employed. Our study performed granular GRADE ratings for each pairwise comparison, revealing that while the quality of evidence for most comparisons was moderate, some reached high quality, and only a few were of low or very low quality. This detailed grading strategy empowers clinicians to make individualized, evidence-based decisions, aligning with the principles of precision medicine.

Despite its comprehensive nature, our network meta-analysis has several limitations that warrant consideration. Firstly, while we aimed to include only full-text publications to ensure the highest methodological rigor, two studies were included based on their conference abstracts: Chen 2025 (24), presented at ASTRO 2025, and Dong 2025 (TALENTACE) (18), presented at the ESMO Gastrointestinal Cancers Congress 2025. We fully acknowledge that the inclusion of studies published solely as abstracts is generally considered a methodological limitation in systematic reviews and meta-analyses, as it potentially impacts the comprehensive evaluation of study methodology and risk of bias. However, our rationale for including these abstracts is multifaceted. For Chen 2025 (24), despite its abstract format, this report highlights clinically meaningful improvements in TTP, OS, ORR, and DCR for radiotherapy combined with toripalimab compared to sorafenib. Crucially, its clear and complete reporting enabled us to extract all necessary quantitative data for our analysis. The abstract details a prospective, open-label, randomized Phase III clinical trial comparing toripalimab plus radiotherapy against sorafenib as first-line treatment in uHCC patients with Vp3 or Vp4 PVTT. Patients presenting with Vp3/Vp4 PVTT in uHCC carry an exceptionally poor prognosis with severely limited treatment options. This study, therefore, offers direct comparative data between an ICIs-based combination and a standard targeted agent within a highly specific, high-risk patient population. Such a comparison is groundbreaking in current clinical practice, and comparable high-quality evidence is scarcely available from other published full-text studies. Excluding this study would invariably create a significant void in our network evidence chain, thereby compromising the comprehensiveness of our assessment of emerging therapeutic regimens in this particular subgroup. Furthermore, while Chen 2025’s report is currently an abstract, it explicitly states its design as a Prospective, Open-Label, Randomized Phase 3 study. This design inherently confers a high level of evidence in evidence-based medicine, and its rigorous methodology lends substantial credibility to the reported results. We have diligently attempted to contact the authors to obtain the full study data but have not yet succeeded. Given the very recent publication year (2025), we surmise that the full publication may not yet be formally available online or could still be undergoing peer review. Similarly, for Dong 2025 (TALENTACE) (18), while also in abstract format, its inclusion is considered indispensable for our network meta-analysis, as it provides crucial, otherwise unavailable, information. This study, a multicenter, randomized Phase III clinical trial, was designed to evaluate the efficacy and safety of on-demand TACE combined with atezolizumab and bevacizumab versus TACE monotherapy as first-line treatment for uHCC patients. This combination therapy strategy represents one of the most cutting-edge and highly anticipated therapeutic directions in the current uHCC landscape, embodying the latest advancements in combining targeted, immune, and interventional treatments. The study reported significantly superior PFS in the combination therapy group compared to TACE monotherapy, which is a positive outcome critically important for assessing the efficacy of such innovative combined regimens. Its inclusion effectively addresses a critical gap in the existing evidence base, thereby comprehensively reflecting the latest progress and inherent complexity within the uHCC treatment paradigm. Similar to Chen 2025 (24), we have also attempted to contact the authors of the TALENTACE study to obtain the full data, but without success to date. Crucially, to address the potential impact of including these abstracts, we performed a sensitivity analysis by excluding both studies and re-running the network meta-analysis. The results of this sensitivity analysis indicated that our primary findings regarding the efficacy hierarchy and effect sizes for key outcomes remained largely consistent. This suggests that the inclusion of these high-impact abstracts did not introduce substantial bias to our overall conclusions. Nevertheless, we acknowledge this as a limitation and encourage future updates of this meta-analysis to incorporate the full-text publications of these and other relevant emerging studies as they become available.

Beyond the specific methodological discussion concerning abstract inclusion, this study also possesses several other inherent limitations that necessitate cautious interpretation. Firstly, due to the intrinsic physical nature of many treatment regimens, most included studies adopted an open-label design, rendering participant and personnel blinding impractical. Studies involving interventional therapies (e.g., HAIC, TACE), in particular, inherently preclude blinding, potentially elevating the risk of performance and detection bias. While 6 studies were rated as ‘some concerns’ due to this, the impact of unblinding on objective endpoints such as OS and PFS is generally considered less substantial than on subjective outcomes. Secondly, direct evidence was limited for some key comparisons, necessitating a primary reliance on indirect evidence. For instance, a notable absence of direct head-to-head comparisons exists between HAIC and immunocombination therapies (e.g., Atezo-Beva). Consequently, our conclusions predominantly rely on indirect evidence established through Sorafenib as a common comparator. This reliance on indirect evidence, particularly when most pairwise comparisons did not reach statistical significance, means that broader inferences beyond the HAIC-Sora vs Sorafenib comparison should be made with increased caution. While consistency checks did not indicate significant inconsistencies, the generalizability of indirect comparisons can still be influenced by subtle differences in study design, patient characteristics, and treatment implementation. Thirdly, heterogeneity in patient population characteristics was evident among the included studies. Geographic distribution showed a predominance of Asian patients (particularly from China), with HBV infection being the main etiology (>80%), whereas studies from Europe and North America often featured higher proportions of HCV and non-viral etiologies. This demographic and etiological variation may compromise the transitivity assumption of treatment effects. Fourthly, significant variations in disease staging across included studies represent another limitation. Immunotherapy trials predominantly enrolled BCLC stage C patients, often with a high proportion of extrahepatic metastasis and major vascular invasion, whereas interventional therapy studies largely focused on BCLC stage B patients. This divergence in patient populations introduces potential for biased effect estimates. Fifthly, adverse event reporting was, in some instances, insufficient and lacked long-term follow-up data, precluding a comprehensive assessment of late toxicities and sustained impacts on quality of life. This is particularly crucial for immunotherapies requiring long-term administration, as certain irAEs (e.g., endocrine dysfunction) may manifest months or even years after treatment cessation (70). Sixth, evidence for certain highly-ranked SUCRA regimens (e.g., DEB-TACE-Apa, TACE-Lenv) originated from a limited number of studies with correspondingly wide CrIs, which inherently limits the precision of their effect estimates and contributed to the lack of statistical significance in many comparisons. The small sample sizes in some of these trials also contributed to the inability to detect statistically significant differences, even for numerically favorable trends. Seventh, the interpretation of ORR findings must account for potential small trial effects. Some comparisons were informed by a limited number of small-sample trials, where few events can lead to unstable effect estimates and exaggerated treatment effects. Imprecision, reflected by wide CrIs that often spanned multiple orders of magnitude, underscores the limited precision of these estimates. Furthermore, heterogeneity in response evaluation across studies remains a concern: included trials varied in imaging frequency, the timing of response assessment, and the adoption of different response criteria (e.g., RECIST v1.1, mRECIST, or investigator-assessed vs. independent review committee-assessed responses). Such methodological heterogeneity may introduce non-negligible variability in ORR estimates across interventions, thereby affecting the reliability of comparative rankings. Consequently, while these regimens demonstrate promising potential, clinical recommendations should be made with caution, pending further validation through high-quality RCTs. Finally, limitations in the granularity of available data precluded a detailed analysis of dose-response relationships or the comprehensive assessment of treatment sequencing strategies. For instance, optimal sequencing patterns for TACE and systemic therapies (e.g., concurrent vs. sequential administration), as well as optimal dosing cycles and duration for ICIs, remain critical yet unaddressed clinical questions.

### 4.6 Future research directions

Based on the findings and limitations of this study, future research endeavors should strategically focus on the following critical areas:Firstly, there is an urgent and pressing need for more high-quality head-to-head RCTs. These trials should directly compare the efficacy and safety of different combination regimens, particularly juxtaposing HAIC-Sora with leading immunocombination regimens, and evaluating interventional therapies against systemic therapy alone.This is especially critical for those regimens that ranked highly in SUCRA but did not achieve statistical significance in pairwise comparisons, as their true comparative efficacy requires direct validation. Such studies must employ rigorous randomization methodologies, achieve adequate sample sizes, and incorporate sufficiently long follow-up periods to generate robust, high-quality direct evidence. Specifically, adequately powered trials are needed to address the small trial effects observed for certain promising regimens, ensuring sufficient statistical power to detect meaningful clinical differences.

Secondly, the establishment of a comprehensive core outcome set (COS) for uHCC treatment is imperative (71). This COS should encompass key indicators such as OS, PFS, ORR, DCR, patient-reported quality of life (QoL), AEs (meticulously classified by type and severity), and relevant health economic evaluation metrics. Beyond standardizing reported outcomes, this also critically includes harmonizing the methods and criteria for response evaluation across trials. Standardizing these outcomes will significantly enhance comparability across diverse studies, thereby facilitating more robust systematic reviews and meta-analyses.

Thirdly, accruing longer-term follow-up data and leveraging real-world evidence (RWE) are essential to comprehensively assess the sustained benefits, potential late toxicities, and long-term impact on quality of life associated with combination therapies. RWE studies, by their nature, can incorporate broader and more heterogeneous patient populations, thus offering invaluable insights into the external validity and generalizability of treatment effects in routine clinical practice (72).

Fourthly, a deeper and more systematic exploration of treatment effect modifiers and predictive biomarkers is warranted. This involves prospectively collecting and rigorously analyzing multi-dimensional biomarker data (e.g., PD-L1 expression, tumor mutational burden, AFP levels, circulating tumor DNA, gut microbiome composition, etc.) to precisely identify which patient subgroups are most likely to derive optimal benefit from specific therapeutic approaches. Concurrently, in-depth mechanistic studies, utilizing advanced techniques such as immunohistochemistry, multi-omics analyses, and relevant animal models, are crucial to elucidate the synergistic mechanisms underlying various combination regimens, thereby providing a robust theoretical foundation for rational treatment design.

Fifthly, refined and granular subgroup network meta-analyses or individual patient data meta-analyses should be undertaken. These analyses would stratify patients based on critical characteristics such as etiological background (HBV vs. HCV vs. non-viral), geographical regions, specific disease stage (e.g., BCLC B vs. C), liver functional status (Child-Pugh A vs. B), and tumor burden (presence or absence of EHS, MVI, or PVI). The ultimate goal of such nuanced analyses is to pinpoint optimal treatment regimens tailored for distinct patient subgroups, thereby advancing the principles of precision medicine in uHCC.

Finally, reinforcing and rigorously adhering to clinical trial reporting standards is paramount. All clinical trials should be prospectively registered on public platforms prior to initiation, and their study design, implementation processes, and comprehensive results must be reported fully and transparently in strict accordance with guidelines such as the CONSORT statement. Furthermore, advocating for open data sharing will foster secondary analyses and enhance evidence synthesis, ultimately accelerating the translation of research findings into improved clinical care.

### 4.7 Conclusion

In summary, this study provides comprehensive and high-quality evidence regarding the efficacy and safety of diverse first-line treatment regimens for uHCC. Drawing upon data from 35 RCTs and 13,595 patients, our network meta-analysis demonstrates with moderate certainty that the HAIC-Sora combination regimen significantly outperforms Sorafenib monotherapy in both OS and PFS, while maintaining an acceptable safety profile. This finding, being the only statistically significant survival benefit observed against sorafenib in our analysis, highlights HAIC-Sora as a robustly supported preferred regimen, particularly for uHCC patients presenting with portal vein invasion or major vascular invasion. Furthermore, HAIC monotherapy exhibited a significant advantage in ORR and demonstrated a favorable safety profile, underscoring its potential as an optimal consideration for balancing efficacy and safety. Other promising combination regimens, including TACE-Lenv, DEB-TACE-Apa, and various immune-angiogenesis combinations, showed encouraging trends towards survival benefits and ranked highly in SUCRA analyses. However, given that these did not achieve statistical significance in pairwise comparisons, and often relied on indirect evidence, the certainty of evidence for these specific regimens warrants further strengthening through additional high-quality direct comparative research.

## Data Availability

The datasets presented in this study can be found in online repositories. The names of the repository/repositories and accession number(s) can be found in the article/**Supplementary Material**.
