# Supplementary material for: First-line therapies for unresectable hepatocellular carcinoma: a network meta-analysis of immune checkpoint inhibitors and transarterial therapies based on 35 randomized trials
Source: Front Oncol. 2026 Apr 29;16:1809232. doi: 10.3389/fonc.2026.1809232 (PMC13167520; doi:10.3389/fonc.2026.1809232)
Supplement: Supplementary file 1 [file DataSheet1.docx]

**First-Line Therapies for Unresectable Hepatocellular Carcinoma: A Network Meta-Analysis of Immune Checkpoint Inhibitors and Transarterial Therapies Based on 35 Randomized Trials**

| **Table of Contents** | | |
| --- | --- | --- |
| Title | Content | page |
| Table S1 | PRISMA NMA Checklist of Items to Include When Reporting a Systematic Review Involving a Network Meta-analysis | 2-5 |
| Table S2 | Literature Search Strategy | 6-7 |
| Table S3 | SUCRA-Based Ranking of Treatment Regimens for Endpoints in the Bayesian Network Meta-Analysis | 8 |
| Table S4 | Model Fit Statistics for Random and Fixed Effects in Bayesian Network Meta-Analysis Across Endpoints | 9 |
| Table S5 | Consistency and Inconsistency Model Fit Comparison and Heterogeneity Assessment Across Endpoints in the Bayesian Network Meta-Analysis | 9 |
| TableS6 | Detailed Quantitative Results of All Included Randomized Controlled Trials | 10-11 |
| Figure S1 | Summary of results from assessment of studies by using Cochrane Risk of Bias Tool 2.0 | 12 |
| Figure S2 | Graph of results from assessment of studies by using Cochrane Risk of Bias Tool 2.0 | 13 |
| Figure S3 | Convergence Diagnostics of Bayesian Network Meta-Analysis for OS | 14 |
| Figure S4 | Convergence Diagnostics of Bayesian Network Meta-Analysis for PFS | 15 |
| Figure S5 | Convergence Diagnostics of Bayesian Network Meta-Analysis for ORR | 16 |
| Figure S6 | Convergence Diagnostics of Bayesian Network Meta-Analysis for ≥3 AEs | 17 |
| Figure S7 | Trace and Posterior Density Plots for Bayesian Network Meta-Analysis of OS | 18 |
| Figure S8 | Trace and Posterior Density Plots for Bayesian Network Meta-Analysis of PFS | 19 |
| Figure S9 | Trace and Posterior Density Plots for Bayesian Network Meta-Analysis of ORR | 20 |
| Figure S10 | Trace and Posterior Density Plots for Bayesian Network Meta-Analysis of ≥3 AEs | 21 |
| Figure S11 | Rank Probability Heatmap of Treatment Regimens for OS | 22 |
| Figure S12 | Rank Probability Heatmap of Treatment Regimens for PFS | 22 |
| Figure S13 | Rank Probability Heatmap of Treatment Regimens for ORR | 22 |
| Figure S14 | Rank Probability Heatmap of Treatment Regimens for ≥3 AEs | 23 |
| Figure S15 | Comparison-adjusted Funnel Plot for OS | 23 |
| Figure S16 | Comparison-adjusted Funnel Plot for PFS | 24 |
| Figure S17 | Comparison-adjusted Funnel Plot for ORR | 24 |
| Figure S18 | Comparison-adjusted Funnel Plot for ≥3 AEs | 25 |

| **Table S1. PRISMA NMA Checklist of Items to Include When Reporting a Systematic Review Involving a Network Meta-analysis** | | | |
| --- | --- | --- | --- |
| **Section/Topic** | **Item #** | **Checklist Item** | **Reported on Page #** |
| **TITLE** |  |  |  |
| Title | 1 | Identify the report as a systematic review *incorporating*  *anetwork meta-analysis (or related form of meta-analysis).* | 1 |
|  |  |  |  |
| **ABSTRACT** |  |  |  |
| Structured summary | 2 | Provide a structured summary including, as applicable:  **Background:** main objectives  **Methods:** data sources; study eligibility criteria, participants, and interventions; study appraisal; and *synthesis methods, such as network meta-analysis.*  **Results:** number of studies and participants identified; summary estimates with corresponding confidence/credible intervals; *treatment rankings may also be discussed. Authors may choose to summarize pairwise comparisons against a chosen treatment included in their analyses for brevity.*  **Discussion/Conclusions:** limitations; conclusions and implications of findings.  **Other:** systematic review registration number with registry name. | 1 |
|  |  |  |  |
| **INTRODUCTION** |  |  |  |
| Rationale | 3 | Describe the rationale for the review in the context of what is already known*, including mention of why a network meta-analysis has been conducted.* | 1-3 |
| Objectives | 4 | Provide an explicit statement of questions being addressed, with reference to participants, interventions, comparisons, outcomes, and study design (PICOS). | 3-4 |
|  |  |  |  |
| **METHODS** |  |  |  |
| Protocol and registration | 5 | Indicate whether a review protocol exists and if and where it can be accessed (e.g., Web address); and, if available, provide registration information, including registration number. | 3 |
| Eligibility criteria | 6 | Specify study characteristics (e.g., PICOS, length of follow-up) and report characteristics (e.g., years considered, language, publication status) used as criteria for eligibility, giving rationale. *Clearly describe eligible treatments included in the treatment network, and note whether any have been clustered or merged into the same node (with justification).* | 3-4, 5, 15 |
| Information sources | 7 | Describe all information sources (e.g., databases with dates of coverage, contact with study authors to identify additional studies) in the search and date last searched. | 3 |
| Search | 8 | Present full electronic search strategy for at least one database, including any limits used, such that it could be repeated. | Supplementary  Table S2 |
| Study selection | 9 | State the process for selecting studies (i.e., screening, eligibility, included in systematic review, and, if applicable, included in the meta-analysis). | 3-4 |
| Data collection process | 10 | Describe method of data extraction from reports (e.g., piloted forms, independently, in duplicate) and any processes for obtaining and confirming data from investigators. | 4 |
| Data items | 11 | List and define all variables for which data were sought (e.g., PICOS, funding sources) and any assumptions and simplifications made. | 3-4 |
| **Geometry of the network** | **S1** | Describe methods used to explore the geometry of the treatment network under study and potential biases related to it. This should include how the evidence base has been graphically summarized for presentation, and what characteristics were compiled and used to describe the evidence base to readers. | 4-5 |
| Risk of bias within individual studies | 12 | Describe methods used for assessing risk of bias of individual studies (including specification of whether this was done at the study or outcome level), and how this information is to be used in any data synthesis. | 4-5 |
| Summary measures | 13 | State the principal summary measures (e.g., risk ratio, difference in means). *Also describe the use of additional summary measures assessed, such as treatment rankings and surface under the cumulative ranking curve (SUCRA) values, as well as modified approaches used to present summary findings from meta-analyses.* | 4-5 |
| Planned methods of analysis | 14 | Describe the methods of handling data and combining results of studies for each network meta-analysis. This should include, but not be limited to:   - *Handling of multi-arm trials;* - *Selection of variance structure;* - *Selection of prior distributions in Bayesian analyses; and* - *Assessment of model fit.* | 4-5 |
| **Assessment of Inconsistency** | **S2** | Describe the statistical methods used to evaluate the agreement of direct and indirect evidence in the treatment network(s) studied. Describe efforts taken to address its presence when found. | 4-5 |
| Risk of bias across studies | 15 | Specify any assessment of risk of bias that may affect the cumulative evidence (e.g., publication bias, selective reporting within studies). | 4-5 |
| Additional analyses | 16 | Describe methods of additional analyses if done, indicating which were pre-specified. This may include, but not be limited to, the following:   - Sensitivity or subgroup analyses; - Meta-regression analyses; - *Alternative formulations of the treatment network; and* - *Use of alternative prior distributions for Bayesian analyses (if applicable).* | NA |
| **RESULTS†** |  |  |  |
| Study selection | 17 | Give numbers of studies screened, assessed for eligibility, and included in the review, with reasons for exclusions at each stage, ideally with a flow diagram. | 5, Figure 1 |
| **Presentation of network structure** | **S3** | Provide a network graph of the included studies to enable visualization of the geometry of the treatment network. | Figure 2 |
| **Summary of network geometry** | **S4** | Provide a brief overview of characteristics of the treatment network. This may include commentary on the abundance of trials and randomized patients for the different interventions and pairwise comparisons in the network, gaps of evidence in the treatment network, and potential biases reflected by the network structure. | 16, 20 |
| Study characteristics | 18 | For each study, present characteristics for which data were extracted (e.g., study size, PICOS, follow-up period) and provide the citations. | Table 1-2 |
| Risk of bias within studies | 19 | Present data on risk of bias of each study and, if available, any outcome level assessment. | Supplementary  Figure S1-2 |
| Results of individual studies | 20 | For all outcomes considered (benefits or harms), present, for each study: 1) simple summary data for each intervention group, and 2) effect estimates and confidence intervals. *Modified approaches may be needed to deal with information from larger networks.* | Supplementary  Table S6 |
| Synthesis of results | 21 | Present results of each meta-analysis done, including confidence/credible intervals. *In larger networks, authors may focus on comparisons versus a particular comparator (e.g. placebo or standard care), with full findings presented in an appendix. League tables and forest plots may be considered to summarize pairwise comparisons.* If additional summary measures were explored (such as treatment rankings), these should also be presented. | Supplementary  Table S6 |
| **Exploration for inconsistency** | **S5** | Describe results from investigations of inconsistency. This may include such information as measures of model fit to compare consistency and inconsistency models, *P* values from statistical tests, or summary of inconsistency estimates from different parts of the treatment network. | Supplementary  Table S5 |
| Risk of bias across studies | 22 | Present results of any assessment of risk of bias across studies for the evidence base being studied. | Supplementary  Figure S1-2 |
| Results of additional analyses | 23 | Give results of additional analyses, if done (e.g., sensitivity or subgroup analyses, meta-regression analyses*, alternative network geometries studied, alternative choice of prior distributions for Bayesian analyses,* and so forth). | NA |
|  |  |  |  |
| **DISCUSSION** |  |  |  |
| Summary of evidence | 24 | Summarize the main findings, including the strength of evidence for each main outcome; consider their relevance to key groups (e.g., healthcare providers, users, and policy-makers). | 21 |
| Limitations | 25 | Discuss limitations at study and outcome level (e.g., risk of bias), and at review level (e.g., incomplete retrieval of identified research, reporting bias). *Comment on the validity of the assumptions, such as transitivity and consistency. Comment on any concerns regarding network geometry (e.g., avoidance of certain comparisons).* | 25-27 |
| Conclusions | 26 | Provide a general interpretation of the results in the context of other evidence, and implications for future research. | 28-29 |
|  |  |  |  |
| **FUNDING** |  |  |  |
| Funding | 27 | Describe sources of funding for the systematic review and other support (e.g., supply of data); role of funders for the systematic review. This should also include information regarding whether funding has been received from manufacturers of treatments in the network and/or whether some of the authors are content experts with professional conflicts of interest that could affect use of treatments in the network. | NA |

| **Table S2. Literature Search Strategy** | |
| --- | --- |
| **Pubmed** | #1 "carcinoma, hepatocellular"[MeSH Terms] 117,879  #2 "Immune Checkpoint Inhibitors"[MeSH Terms] 17,805  #3 "chemoembolization, therapeutic"[MeSH Terms] 8,231  #4 "carcinomas hepatocellular"[Title/Abstract] OR "hepatocellular carcinomas"[Title/Abstract] OR "hepatocellular carcinoma"[Title/Abstract] OR "Hepatoma"[Title/Abstract] OR "Hepatomas"[Title/Abstract] OR (("Liver"[MeSH Terms] OR "Liver"[All Fields] OR "livers"[All Fields] OR "liver s"[All Fields]) AND "cancer adult"[Title/Abstract]) OR "adult liver cancer"[Title/Abstract] OR "adult liver cancers"[Title/Abstract] OR (("cancer s"[All Fields] OR "cancerated"[All Fields] OR "canceration"[All Fields] OR "cancerization"[All Fields] OR "cancerized"[All Fields] OR "cancerous"[All Fields] OR "Neoplasms"[MeSH Terms] OR "Neoplasms"[All Fields] OR "Cancer"[All Fields] OR "Cancers"[All Fields]) AND "adult liver"[Title/Abstract]) OR (("cancer s"[All Fields] OR "cancerated"[All Fields] OR "canceration"[All Fields] OR "cancerization"[All Fields] OR "cancerized"[All Fields] OR "cancerous"[All Fields] OR "Neoplasms"[MeSH Terms] OR "Neoplasms"[All Fields] OR "Cancer"[All Fields] OR "Cancers"[All Fields]) AND "adult liver"[Title/Abstract]) OR (("Liver"[MeSH Terms] OR "Liver"[All Fields] OR "livers"[All Fields] OR "liver s"[All Fields]) AND "cancers adult"[Title/Abstract]) OR "liver cell carcinoma"[Title/Abstract] OR "carcinoma liver cell"[Title/Abstract] OR (("Carcinoma"[MeSH Terms] OR "Carcinoma"[All Fields] OR "Carcinomas"[All Fields] OR "carcinoma s"[All Fields]) AND "liver cell"[Title/Abstract]) OR "cell carcinoma liver"[Title/Abstract] OR "cell carcinomas liver"[Title/Abstract] OR "liver cell carcinomas"[Title/Abstract] OR (("carcinoma, hepatocellular"[MeSH Terms] OR ("Carcinoma"[All Fields] AND "Hepatocellular"[All Fields]) OR "hepatocellular carcinoma"[All Fields] OR ("Liver"[All Fields] AND "Cell"[All Fields] AND "Carcinoma"[All Fields]) OR "liver cell carcinoma"[All Fields]) AND "Adult"[Title/Abstract]) OR "liver neoplasms"[Title/Abstract] OR "hepatic neoplasm"[Title/Abstract] OR "HCC"[Title/Abstract] 181,444  #5 "checkpoint inhibitors immune"[Title/Abstract] OR "immune checkpoint blockers"[Title/Abstract] OR "checkpoint blockers immune"[Title/Abstract] OR "immune checkpoint inhibitor"[Title/Abstract] OR "checkpoint inhibitor immune"[Title/Abstract] OR "immune checkpoint blockade"[Title/Abstract] OR "checkpoint blockade immune"[Title/Abstract] OR "immune checkpoint inhibition"[Title/Abstract] OR "checkpoint inhibition immune"[Title/Abstract] OR "pd 1 pd l1 blockade"[Title/Abstract] OR "blockade pd 1 pd l1"[Title/Abstract] OR "pd 1 pd l1 blockade"[Title/Abstract] OR "pd 1 inhibitors"[Title/Abstract] OR "pd 1 inhibitors"[Title/Abstract] OR "programmed cell death protein 1 inhibitor"[Title/Abstract] OR "programmed cell death protein 1 inhibitors"[Title/Abstract] OR "pd 1 inhibitor"[Title/Abstract] OR "inhibitor pd 1"[Title/Abstract] OR "pd 1 inhibitor"[Title/Abstract] OR "Nivolumab"[Title/Abstract] OR "Pembrolizumab"[Title/Abstract] OR "Tislelizumab"[Title/Abstract] OR "Sintilimab"[Title/Abstract] OR "Camrelizumab"[Title/Abstract] OR "Toripalimab"[Title/Abstract] OR "Cemiplimab"[Title/Abstract] OR "Finotonlimab"[Title/Abstract] OR "serplulimab"[Title/Abstract] OR "Cemiplimab"[Title/Abstract] OR "Spartalizumab"[Title/Abstract] OR "pd l1 inhibitors"[Title/Abstract] OR "pd l1 inhibitors"[Title/Abstract] OR "programmed death ligand 1 inhibitors"[Title/Abstract] OR "programmed death ligand 1 inhibitors"[Title/Abstract] OR "pd l1 inhibitor"[Title/Abstract] OR "pd l1 inhibitor"[Title/Abstract] OR "Atezolizumab"[Title/Abstract] OR "Durvalumab"[Title/Abstract] OR "avelumab"[Title/Abstract] OR "ctla 4 inhibitors"[Title/Abstract] OR "ctla 4 inhibitors"[Title/Abstract] OR "cytotoxic t lymphocyte associated protein 4 inhibitors"[Title/Abstract] OR "cytotoxic t lymphocyte associated protein 4 inhibitors"[Title/Abstract] OR "cytotoxic t lymphocyte associated protein 4 inhibitor"[Title/Abstract] OR "cytotoxic t lymphocyte associated protein 4 inhibitor"[Title/Abstract] OR "ctla 4 inhibitor"[Title/Abstract] OR "ctla 4 inhibitor"[Title/Abstract] OR "Ipilimumab"[Title/Abstract] OR "Tremelimumab"[Title/Abstract] OR "Cadonilimab"[Title/Abstract] OR "programmed death 1 inhibitor"[Title/Abstract] OR "anti pd 1"[Title/Abstract] OR "Anti-PD-L1"[Title/Abstract] OR "anti pd 1 pd l1"[Title/Abstract] OR "Immunotherapy"[Title/Abstract] OR "immune related therapy"[Title/Abstract] 209,789  #6 #3 OR "transarterial chemoembolization"[Title/Abstract] OR "TACE"[Title/Abstract] OR "hepatic arterial infusion chemotherapy"[Title/Abstract] OR "HAIC"[Title/Abstract] 14,934  #7 #1 OR #4 197,883  #8 #2 OR #5 212,313  #9 #6 OR #8 226,094  #10 #9 AND #7 AND (randomizedcontrolledtrial[Filter]) 368 |
| **Web of Science** | #1 TS=("carcinoma, hepatocellular" OR "carcinomas hepatocellular" OR "hepatocellular carcinomas" OR "hepatocellular carcinoma" OR "Hepatoma" OR "Hepatomas" OR (("Liver" OR "Liver" OR "livers" OR "liver s") AND "cancer adult") OR "adult liver cancer" OR "adult liver cancers" OR (("cancer s" OR "cancerated" OR "canceration" OR "cancerization" OR "cancerized" OR "cancerous" OR "Neoplasms" OR "Neoplasms" OR "Cancer" OR "Cancers") AND "adult liver") OR (("cancer s" OR "cancerated" OR "canceration" OR "cancerization" OR "cancerized" OR "cancerous" OR "Neoplasms" OR "Neoplasms" OR "Cancer" OR "Cancers") AND "adult liver") OR (("Liver" OR "Liver" OR "livers" OR "liver s") AND "cancers adult") OR "liver cell carcinoma" OR "carcinoma liver cell" OR (("Carcinoma" OR "Carcinoma" OR "Carcinomas" OR "carcinoma s") AND "liver cell") OR "cell carcinoma liver" OR "cell carcinomas liver" OR "liver cell carcinomas" OR (("carcinoma, hepatocellular" OR ("Carcinoma" AND "Hepatocellular") OR "hepatocellular carcinoma" OR ("Liver" AND "Cell" AND "Carcinoma") OR "liver cell carcinoma") AND "Adult") OR "liver neoplasms" OR "hepatic neoplasm" OR "HCC") 183035  #2 TS=("checkpoint inhibitors immune" OR "immune checkpoint blockers" OR "checkpoint blockers immune" OR "immune checkpoint inhibitor" OR "checkpoint inhibitor immune" OR "immune checkpoint blockade" OR "checkpoint blockade immune" OR "immune checkpoint inhibition" OR "checkpoint inhibition immune" OR "pd 1 pd l1 blockade" OR "blockade pd 1 pd l1" OR "pd 1 pd l1 blockade" OR "pd 1 inhibitors" OR "pd 1 inhibitors" OR "programmed cell death protein 1 inhibitor" OR "programmed cell death protein 1 inhibitors" OR "pd 1 inhibitor" OR "inhibitor pd 1" OR "pd 1 inhibitor" OR "Nivolumab" OR "Pembrolizumab" OR "Tislelizumab" OR "Sintilimab" OR "Camrelizumab" OR "Toripalimab" OR "Cemiplimab" OR "Finotonlimab" OR "serplulimab" OR "Cemiplimab" OR "Spartalizumab" OR "pd l1 inhibitors" OR "pd l1 inhibitors" OR "programmed death ligand 1 inhibitors" OR "programmed death ligand 1 inhibitors" OR "pd l1 inhibitor" OR "pd l1 inhibitor" OR "Atezolizumab" OR "Durvalumab" OR "avelumab" OR "ctla 4 inhibitors" OR "ctla 4 inhibitors" OR "cytotoxic t lymphocyte associated protein 4 inhibitors" OR "cytotoxic t lymphocyte associated protein 4 inhibitors" OR "cytotoxic t lymphocyte associated protein 4 inhibitor" OR "cytotoxic t lymphocyte associated protein 4 inhibitor" OR "ctla 4 inhibitor" OR "ctla 4 inhibitor" OR "Ipilimumab" OR "Tremelimumab" OR "Cadonilimab" OR "programmed death 1 inhibitor" OR "anti pd 1" OR "Anti-PD-L1" OR "anti pd 1 pd l1" OR "Immunotherapy" OR "immune related therapy") 234156  #3 TS=("chemoembolization, therapeutic" OR "transarterial chemoembolization" OR "TACE" OR "hepatic arterial infusion chemotherapy" OR "HAIC") 12475  #4 #2 OR #3 245277  #5 #1 AND #4 20318  #6 TS=("Randomized Controlled Trial" OR RCT OR "Randomized Controlled Trials") 319276  #7 #5 AND #6 1009 |
| **Cochrane** | #1 MeSH descriptor: [Carcinoma, Hepatocellular] explode all trees 2876  #2 MeSH descriptor: [Immune Checkpoint Inhibitors] explode all trees 437  #3 MeSH descriptor: [Chemoembolization, Therapeutic] explode all trees 499  #4 "Carcinoma, Hepatocellular" OR "Carcinomas, Hepatocellular" OR "Hepatocellular Carcinomas" OR "Hepatocellular Carcinoma" OR Hepatoma OR Hepatomas OR "Liver Cancer, Adult" OR "Adult Liver Cancer" OR "Adult Liver Cancers" OR "Cancer, Adult Liver" OR "Cancers, Adult Liver" OR "Liver Cancers, Adult" OR "Liver Cell Carcinoma" OR "Carcinoma, Liver Cell" OR "Carcinomas, Liver Cell" OR "Cell Carcinoma, Liver" OR "Cell Carcinomas, Liver" OR "Liver Cell Carcinomas" OR "Liver Cell Carcinoma, Adult" OR "Liver Neoplasms" OR "Hepatic Neoplasm" OR HCC 9904  #5 "Immune Checkpoint Inhibitors" OR "Checkpoint Inhibitors, Immune" OR "Immune Checkpoint Blockers" OR "Checkpoint Blockers, Immune" OR "Immune Checkpoint Inhibitor" OR "Checkpoint Inhibitor, Immune" OR "Immune Checkpoint Blockade" OR "Checkpoint Blockade, Immune" OR "Immune Checkpoint Inhibition" OR "Checkpoint Inhibition, Immune" OR "PD-1-PD-L1 Blockade" OR "Blockade, PD-1-PD-L1" OR "PD 1 PD L1 Blockade" OR "PD-1 Inhibitors" OR "PD 1 Inhibitors" OR "Programmed Cell Death Protein 1 Inhibitor" OR "Programmed Cell Death Protein 1 Inhibitors" OR "PD-1 Inhibitor" OR "Inhibitor, PD-1" OR "PD 1 Inhibitor" OR Nivolumab OR Pembrolizumab OR Tislelizumab OR Sintilimab OR Camrelizumab OR Toripalimab OR Cemiplimab OR Finotonlimab OR serplulimab OR Spartalizumab OR "PD-L1 Inhibitors" OR "PD L1 Inhibitors" OR "Programmed Death-Ligand 1 Inhibitors" OR "Programmed Death Ligand 1 Inhibitors" OR "PD-L1 Inhibitor" OR "PD L1 Inhibitor" OR Atezolizumab OR Durvalumab OR avelumab OR "CTLA-4 Inhibitors" OR "CTLA 4 Inhibitors" OR "Cytotoxic T-Lymphocyte-Associated Protein 4 Inhibitors" OR "Cytotoxic T Lymphocyte Associated Protein 4 Inhibitors" OR "Cytotoxic T-Lymphocyte-Associated Protein 4 Inhibitor" OR "Cytotoxic T Lymphocyte Associated Protein 4 Inhibitor" OR "CTLA-4 Inhibitor" OR "CTLA 4 Inhibitor" OR Ipilimumab OR Tremelimumab OR Cadonilimab OR "Programmed Death 1 Inhibitor" OR "Anti-PD-1" OR "Anti-PD-L1" OR "anti-PD-1/PD-L1" OR Immunotherapy OR "immune-related therapy" 27230  #6 #1 OR #4 9904  #7 #2 OR #5 27230  #8 #3 OR "transarterial chemoembolization" OR "TACE" OR "hepatic arterial infusion chemotherapy" OR "HAIC" 2071  #9 #7 OR #8 29045  #10 #9 AND #6 2789  #11 "Randomized Controlled Trial" OR RCT OR "Randomized Controlled Trials" 795483  #12 #10 AND #11 1183 |
| **Embase** | #1 'liver cell carcinoma'/exp OR 'liver cell carcinoma' OR 'liver cancer'/exp OR 'liver cancer' OR 'cancer of the liver':ti,ab,kw OR 'cancer, liver':ti,ab,kw OR 'carcinomatous liver':ti,ab,kw OR 'hepatic cancer':ti,ab,kw OR 'hepatic malignancies':ti,ab,kw OR 'hepatic malignancy':ti,ab,kw OR 'hepatocellular cancer':ti,ab,kw OR 'hepatocellular carcinomatosis':ti,ab,kw OR 'hepatocellular malignancies':ti,ab,kw OR 'hepatocellular malignancy':ti,ab,kw OR 'hepatocyte cancer':ti,ab,kw OR 'liver cell cancer':ti,ab,kw OR 'liver malignancies':ti,ab,kw OR 'liver malignancy':ti,ab,kw OR 'liver primary cancer':ti,ab,kw OR 'malignancies of the liver':ti,ab,kw OR 'malignancy of the liver':ti,ab,kw OR 'malignant hepatic neoplasm':ti,ab,kw OR 'malignant hepatic tumor':ti,ab,kw OR 'malignant hepatic tumour':ti,ab,kw OR 'malignant liver neoplasm':ti,ab,kw OR 'malignant liver tumor':ti,ab,kw OR 'malignant liver tumour':ti,ab,kw OR 'malignant neoplasm of the liver':ti,ab,kw OR 'malignant neoplasms of the liver':ti,ab,kw OR 'malignant tumor of the liver':ti,ab,kw OR 'malignant tumors of the liver':ti,ab,kw OR 'malignant tumour of the liver':ti,ab,kw OR 'malignant tumours of the liver':ti,ab,kw OR 'primary liver cancer':ti,ab,kw 407009  #2 'immune checkpoint inhibitor'/exp OR 'immune checkpoint blocker':ti,ab,kw OR 'immune checkpoint inhibitors':ti,ab,kw OR 'immune checkpoint blockers':ti,ab,kw OR 'immune checkpoint inhibitor':ti,ab,kw OR 'immune checkpoint blockade':ti,ab,kw OR 'immune checkpoint inhibition':ti,ab,kw OR 'pd-1-pd-l1 blockade':ti,ab,kw OR 'pd 1 pd l1 blockade':ti,ab,kw OR 'pd-1 inhibitors':ti,ab,kw OR 'pd 1 inhibitors':ti,ab,kw OR 'programmed cell death protein 1 inhibitor':ti,ab,kw OR 'programmed cell death protein 1 inhibitors':ti,ab,kw OR 'pd-1 inhibitor':ti,ab,kw OR 'pd 1 inhibitor':ti,ab,kw OR 'nivolumab':ti,ab,kw OR 'pembrolizumab':ti,ab,kw OR 'tislelizumab':ti,ab,kw OR 'sintilimab':ti,ab,kw OR 'camrelizumab':ti,ab,kw OR 'toripalimab':ti,ab,kw OR 'cemiplimab':ti,ab,kw OR 'finotonlimab':ti,ab,kw OR 'serplulimab':ti,ab,kw OR 'spartalizumab':ti,ab,kw OR 'pd-l1 inhibitors':ti,ab,kw OR 'pd l1 inhibitors':ti,ab,kw OR 'programmed death-ligand 1 inhibitors':ti,ab,kw OR 'programmed death ligand 1 inhibitors':ti,ab,kw OR 'pd-l1 inhibitor':ti,ab,kw OR 'pd l1 inhibitor':ti,ab,kw OR 'atezolizumab':ti,ab,kw OR 'durvalumab':ti,ab,kw OR 'avelumab':ti,ab,kw OR 'ctla-4 inhibitors':ti,ab,kw OR 'ctla 4 inhibitors':ti,ab,kw OR 'cytotoxic t-lymphocyte-associated protein 4 inhibitors':ti,ab,kw OR 'cytotoxic t lymphocyte associated protein 4 inhibitors':ti,ab,kw OR 'cytotoxic t-lymphocyte-associated protein 4 inhibitor':ti,ab,kw OR 'cytotoxic t lymphocyte associated protein 4 inhibitor':ti,ab,kw OR 'ctla-4 inhibitor':ti,ab,kw OR 'ctla 4 inhibitor':ti,ab,kw OR 'ipilimumab':ti,ab,kw OR 'tremelimumab':ti,ab,kw OR 'cadonilimab':ti,ab,kw OR 'programmed death 1 inhibitor':ti,ab,kw OR 'anti-pd-1':ti,ab,kw OR 'anti-pd-l1':ti,ab,kw OR 'anti-pd-1/pd-l1':ti,ab,kw OR 'immunotherapy':ti,ab,kw OR 'immune-related therapy':ti,ab,kw 369030  #3 'arterial chemoembolization'/exp OR 'arterial chemoembolization' OR 'transarterial chemoembolization':ti,ab,kw OR 'tace':ti,ab,kw OR 'hepatic arterial infusion chemotherapy':ti,ab,kw OR 'haic':ti,ab,kw 23931  #4 #2 OR #3 390,559  #5 #1 AND #4 43406  #6 #5 AND 'randomized controlled trial'/de 2072 |

| **Table S3. SUCRA-Based Ranking of Treatment Regimens for Endpoints in the Bayesian Network Meta-Analysis** | | | | | | | | |
| --- | --- | --- | --- | --- | --- | --- | --- | --- |
| **Treatment** | **OS** | | **PFS** | | **ORR** | | **≥3 AEs** | |
|  | SUCRA(%) | Rank | SUCRA(%) | Rank | SUCRA(%) | Rank | SUCRA(%) | Rank |
| Tira-Atezo-Beva | 71.7 | 4 | 65.3 | 5 | 57.6 | 10 | 54.8 | 11 |
| TACE-Atezo-Beva | - | - | 45.0 | 17 | - | - | 46.8 | 14 |
| RT-Tori | - | - | - | - | 63.9 | 8 | - | - |
| Durva-Trem | 43.5 | 15 | - | - | 47.1 | 16 | 37.1 | 18 |
| Camre-Rivo | 55.5 | 11 | 63.6 | 7 | 50.6 | 14 | 17.0 | 25 |
| Anlo-Penpu | 49.3 | 13 | 63.1 | 8 | 39.1 | 21 | 59.0 | 10 |
| Fino-SCT510 | 57.8 | 9 | 65.2 | 6 | 66.4 | 6 | 39.7 | 16 |
| Tori-Beva | 43.3 | 16 | 47.4 | 14 | 50.0 | 15 | 72.0 | 5 |
| Atezo-Cabo | 29.0 | 23 | 43.4 | 18 | 38.7 | 22 | 28.4 | 21 |
| Tisle | 36.5 | 20 | 22.1 | 26 | 36.7 | 23 | 91.3 | 1 |
| Sinti-IBI305 | 60.2 | 7 | 59.4 | 9 | 54.0 | 13 | 51.6 | 12 |
| Pembro-Lenv | 40.0 | 17 | 58.6 | 10 | 46.4 | 17 | 42.2 | 15 |
| Atezo-Beva | 51.2 | 12 | 50.2 | 11 | 39.1 | 20 | 64.0 | 8 |
| Nivo | 37.0 | 18 | 30.3 | 22 | 32.5 | 25 | 89.0 | 3 |
| TACE-Durva-Beva | - | - | 40.9 | 19 | 68.0 | 4 | 23.6 | 23 |
| Lenv-Pembro  -TACE | 47.3 | 14 | 49.4 | 12 | 67.6 | 5 | 22.3 | 24 |
| TACE-Lenv | 71.7 | 3 | 85.5 | 1 | 57.1 | 11 | 16.7 | 26 |
| HAIC | 73.5 | 2 | 65.7 | 4 | 84.2 | 1 | 90.8 | 2 |
| HAIC-Sora | 63.0 | 6 | 73.2 | 3 | 58.7 | 9 | 38.9 | 17 |
| ISP-TACE | 67.8 | 5 | - | - | 41.9 | 19 | 33.5 | 19 |
| DEB-TACE | 56.7 | 10 | 46.4 | 16 | 71.2 | 3 | - | - |
| DEB-TACE-Apa | 82.7 | 1 | 74.1 | 2 | - | - | - | - |
| Lenv | 27.4 | 24 | 49.2 | 13 | 33.0 | 24 | 50.1 | 13 |
| TACE-Sora | 33.4 | 21 | 39.2 | 21 | 21.7 | 27 | 25.4 | 22 |
| DEB-TACE-Sora | 60.2 | 8 | 47.1 | 15 | 72.6 | 2 | - | - |
| TACE | 32.3 | 22 | 22.8 | 24 | 56.9 | 12 | 74.0 | 4 |
| Sora | 22.4 | 25 | 22.2 | 25 | 11.2 | 28 | 63.5 | 9 |
| Durva | 36.7 | 19 | - | - | 42.3 | 18 | 66.9 | 7 |
| TACE-Durva | - | - | 30.0 | 23 | 66.1 | 7 | 69.7 | 6 |
| Cabo | - | - | 40.7 | 20 | 25.4 | 26 | 31.7 | 20 |

| **Table S4. Model Fit Statistics for Random and Fixed Effects in Bayesian Network Meta-Analysis Across Endpoints** | | | | |
| --- | --- | --- | --- | --- |
| **Endpoints** | **Model Type** | **Dbar** | **pD** | **DIC** |
| OS | Random effect | 33.55 | 31.41 | 64.96 |
|  | Fixed effect | 33.52 | 31.32 | 64.84 |
| PFS | Random effect | 32.43 | 31.07 | 63.50 |
|  | Fixed effect | 32.33 | 31.01 | 63.34 |
| ORR | Random effect | 65.85 | 64.07 | 129.92 |
|  | Fixed effect | 65.82 | 64.09 | 129.91 |
| ≥3 AEs | Random effect | 53.51 | 52.24 | 105.75 |
|  | Fixed effect | 53.53 | 52.27 | 105.80 |

| **Table S5. Consistency and Inconsistency Model Fit Comparison and Heterogeneity Assessment Across Endpoints in the Bayesian Network Meta-Analysis** | | | | | |
| --- | --- | --- | --- | --- | --- |
| **Endpoints** | **Model Type** | **Dbar** | **pD** | **DIC** | **I^2^** |
| OS | Consistency | 33.55 | 31.41 | 64.96 | 5% |
|  | Inconsistency | 33.27 | 32.28 | 65.55 | 4% |
| PFS | Consistency | 32.43 | 31.07 | 63.50 | 4% |
|  | Inconsistency | 32.26 | 31.50 | 63.76 | 4% |
| ORR | Consistency | 65.85 | 64.07 | 129.92 | 3% |
|  | Inconsistency | 65.53 | 63.43 | 128.96 | 2% |
| ≥3 AEs | Consistency | 53.51 | 52.24 | 105.75 | 3% |
|  | Inconsistency | 52.65 | 52.04 | 104.69 | 1% |

| **Table S6**. Detailed Quantitative Results of All Included Randomized Controlled Trials | | | | | | | | | | |
| --- | --- | --- | --- | --- | --- | --- | --- | --- | --- | --- |
| Study | OS | | | PFS | | | ORR | | ≥3AEs | |
|  | HR | LL | UL | HR | LL | UL | Intervention  arm(n/N) | Control  arm(n/N) | Intervention  arm(n/N) | Control  arm(n/N) |
| Chen 2025 | 0.120 | 0.032 | 0.447 | - | - | - | 11/25 | 1/11 | - | - |
| Cheng 2022  (IMbrave150) | 0.660 | 0.520 | 0.850 | 0.650 | 0.530 | 0.810 | 97/326 | 18/159 | 149/329 | 73/156 |
| Ding 2021 | 0.600 | 0.290 | 1.260 | - | - | - | 17/32 | 8/32 | 18/32 | 14/32 |
| Dong 2025  (TALENTACE) | - | - | - | 0.710 | 0.550 | 0.920 | - | - | 109/171 | 72/171 |
| Duan 2024 | 0.526 | 0.371 | 0.744 | 0.564 | 0.435 | 0.733 | - | - | - | - |
| Finn 2025  (IMbrave152) | 0.940 | 0.720 | 1.220 | 0.970 | 0.800 | 1.170 | 99/331 | 88/338 | 147/332 | 122/333 |
| Finn 2025  (MORPHEUS  -Liver) | 0.390 | 0.190 | 0.780 | 0.510 | 0.270 | 0.950 | 17/40 | 2/18 | 8/40 | 4/18 |
| He 2019  (SoraHAIC) | 0.350 | 0.260 | 0.480 | 0.330 | 0.250 | 0.430 | 51/125 | 3/122 | 66/124 | 51/121 |
| Ikeda 2016 | 0.600 | 0.380 | 0.960 | 0.780 | 0.520 | 1.160 | 13/60 | 3/41 | - | - |
| Kondo 2019  (SCOOP-2) | 1.080 | 0.630 | 1.860 | - | - | - | 5/35 | 3/33 | - | - |
| Kudo 2018  (SILIUS) | 1.009 | 0.743 | 1.371 | 0.753 | 0.566 | 1.003 | 37/102 | 18/103 | - | - |
| Kudo 2018  (REFLECT) | 0.920 | 0.790 | 1.060 | 0.660 | 0.570 | 0.770 | 115/478 | 44/476 | 270/476 | 231/475 |
| Kudo 2022  (TACTICS) | 0.861 | 0.607 | 1.223 | 0.661 | 0.466 | 0.938 | - | - | - | - |
| Kudo 2024  (LEAP-012) | 0.800 | 0.570 | 1.110 | 0.660 | 0.510 | 0.840 | 111/237 | 80/243 | 169/237 | 76/241 |
| Lai 2025  (SHATA-001) | 0.580 | 0.420 | 0.800 | 0.480 | 0.350 | 0.650 | 66/141 | 7/66 | 55/140 | 34/60 |
| Lau 2025  (HIMALAYA) | 0.76 | 0.650 | 0.890 | - | - | - | 79/393 | 20/389 | 68/388 | 37/374 |
|  | 0.85 | 0.730 | 1.000 | - | - | - | 66/389 | 20/389 | 33/388 | 37/374 |
| Li 2022 | 0.580 | 0.450 | 0.750 | 0.570 | 0.450 | 0.720 | 73/159 | 28/156 | 78/157 | 107/155 |
| Llovet 2023  (Leap-002) | 0.800 | 0.570 | 1.110 | 0.660 | 0.510 | 0.840 | 111/237 | 80/243 | 169/237 | 76/241 |
| Lu 2023 | 0.560 | 0.350 | 0.900 | - | - | - | 29/50 | 19/50 | 16/49 | 18/50 |
| Lyu 2022  (FOHAIC-1) | 0.408 | 0.301 | 0.553 | 0.451 | 0.340 | 0.598 | 41/130 | 2/132 | 26/128 | 62/129 |
| Meyer 2017  (TACE 2) | 0.910 | 0.670 | 1.240 | 0.990 | 0.770 | 1.270 | 56/157 | 49/156 | - | - |
| Park 2019  (STAH) | 0.910 | 0.687 | 1.205 | 0.730 | 0.589 | 0.912 | 20/170 | 10/169 | 51/153 | 33/167 |
| Peng 2023  (LAUNCH) | 0.450 | 0.330 | 0.610 | 0.430 | 0.340 | 0.550 | 78/170 | 35/168 | - | - |
| Qin 2023  (RATIONALE  -301) | 0.850 | 0.710 | 1.020 | 1.110 | 0.920 | 1.330 | 49/342 | 18/332 | 75/338 | 173/324 |
| Qin 2025  (CARES-310) | 0.620 | 0.490 | 0.800 | 0.520 | 0.410 | 0.650 | 69/272 | 16/271 | 220/272 | 141/269 |
| Ren 2021  (ORIENT-32) | 0.570 | 0.430 | 0.750 | 0.560 | 0.460 | 0.700 | 75/365 | 7/172 | 209/380 | 89/185 |
| Sangro 2025  (EMERALD-1) | - | - | - | 0.77 | 0.610 | 0.980 | 88/202 | 60/203 | 41/154 | 12/200 |
|  | - | - | - | 0.94 | 0.750 | 1.190 | 84/205 | 60/203 | 15/232 | 12/200 |
| Shi 2023 | 0.658 | 0.395 | 1.087 | 0.752 | 0.498 | 1.136 | - | - | - | - |
| Shi 2025  (HEPATORCH) | 0.760 | 0.580 | 0.990 | 0.690 | 0.530 | 0.910 | 41/162 | 10/164 | 124/162 | 135/164 |
| Yau 2022  (CheckMate459) | 0.850 | 0.720 | 1.020 | 0.930 | 0.790 | 1.100 | 57/371 | 26/372 | 82/367 | 180/363 |
| Yau 2024  (COSMIC-312) | 0.98 | 0.780 | 1.240 | 0.740 | 0.560 | 0.970 | 56/432 | 10/217 | 340/429 | 125/207 |
|  | - | - | - | 0.78 | 0.560 | 1.090 | 14/188 | 10/217 | 146/188 | 125/207 |
| Zhao 2025 | 0.600 | 0.440 | 0.810 | 0.500 | 0.380 | 0.650 | 76/229 | 5/116 | 121/230 | 44/116 |
| Zheng 2022 | 0.280 | 0.150 | 0.530 | 0.260 | 0.150 | 0.470 | 13/32 | 1/32 | 19/32 | 8/32 |
| Zhou 2024 | 0.690 | 0.480 | 0.980 | 0.630 | 0.420 | 0.950 | 51/77 | 36/77 | - | - |
| Zhou 2025  (APOLLO) | 0.690 | 0.550 | 0.870 | 0.520 | 0.410 | 0.660 | 89/433 | 16/216 | 215/432 | 101/211 |
| HR, Hazard Ratio; LL, 95% CrI Lower Limit; UL, 95% CrI Upper Limit; n = number of responders, N = total number of participants | | | | | | | | | | |


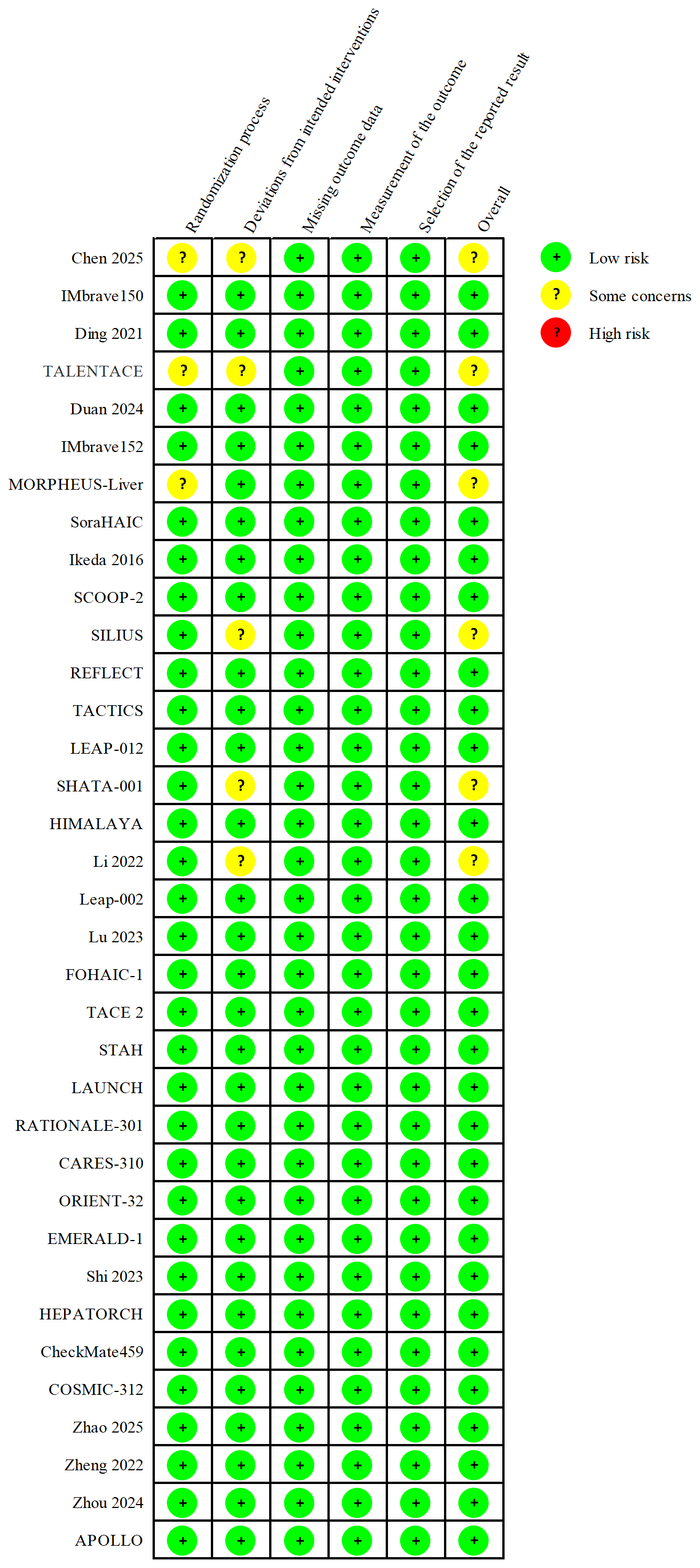


**Figure S1. Summary of results from assessment of studies by using Cochrane Risk of Bias Tool 2.0**

Studies were classified into one of three categories: low, high risk or having “some concerns”


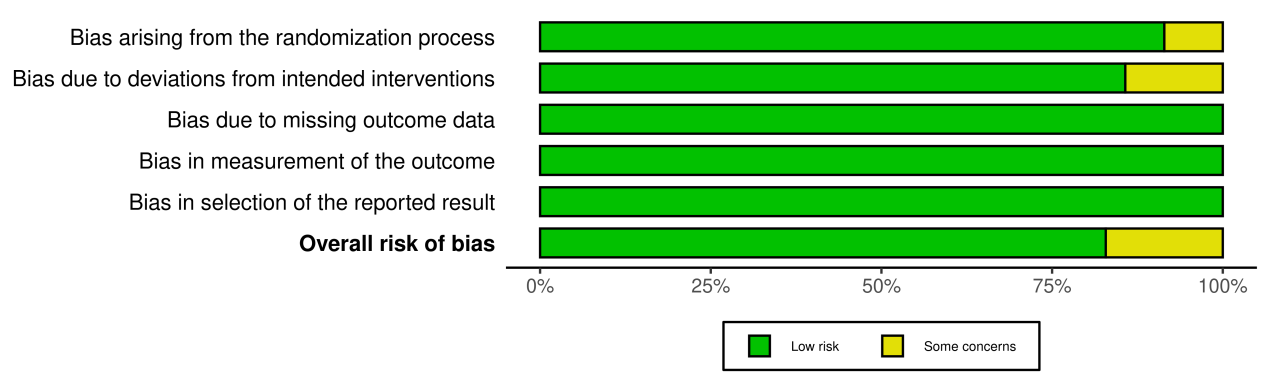


**Figure S2. Graph of results from assessment of studies by using Cochrane Risk of Bias Tool 2.0**

| **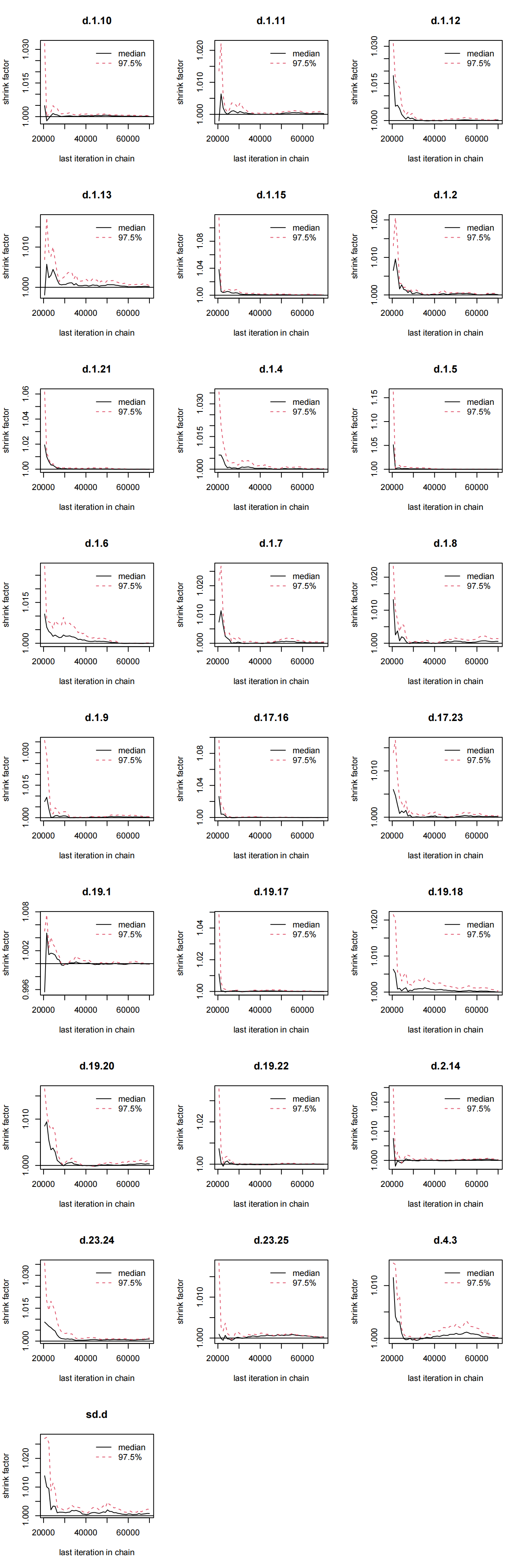** |
| --- |
| **Figure S3. Convergence Diagnostics of Bayesian Network Meta-Analysis for OS** |

| **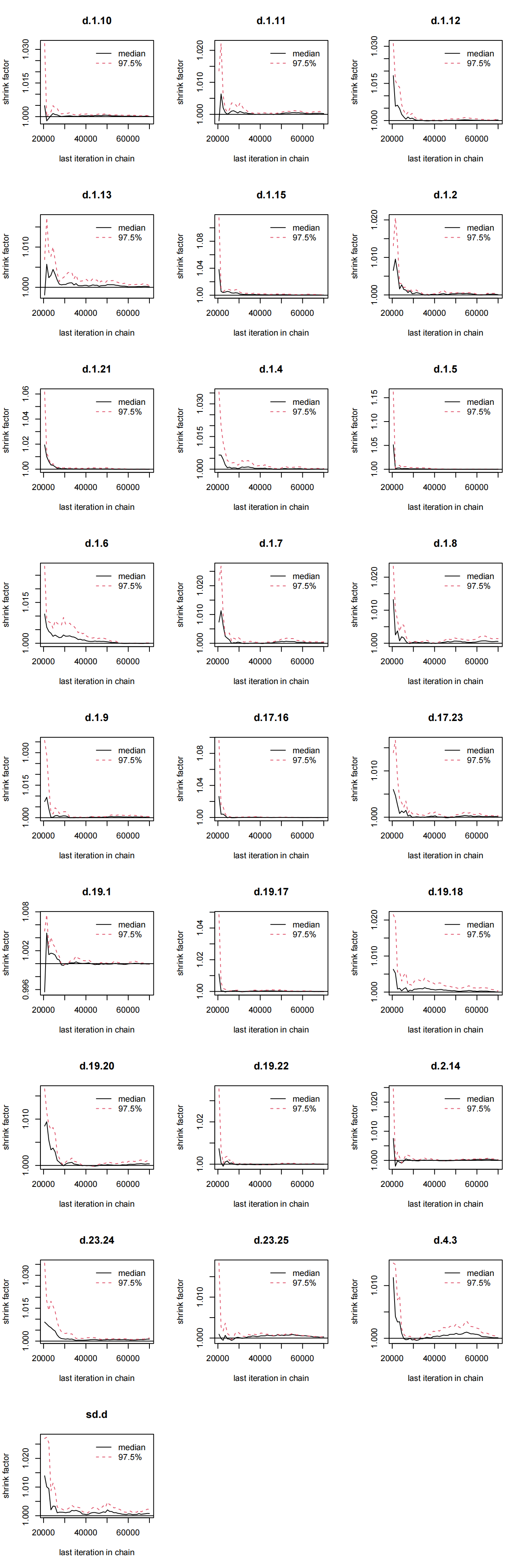** |
| --- |
| **Figure S4. Convergence Diagnostics of Bayesian Network Meta-Analysis for PFS** |

| **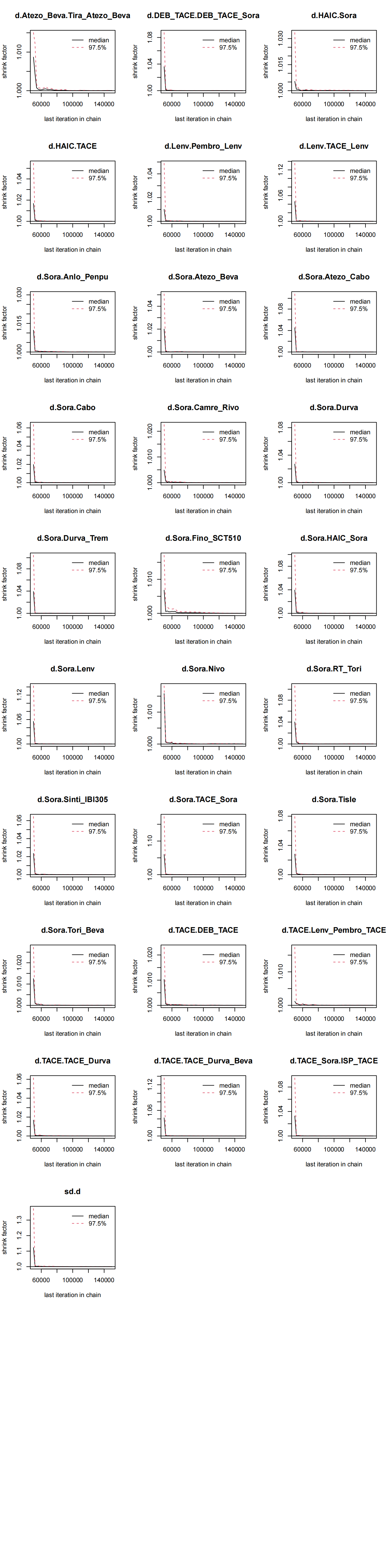** |
| --- |
| **Figure S5. Convergence Diagnostics of Bayesian Network Meta-Analysis for ORR** |

| \| **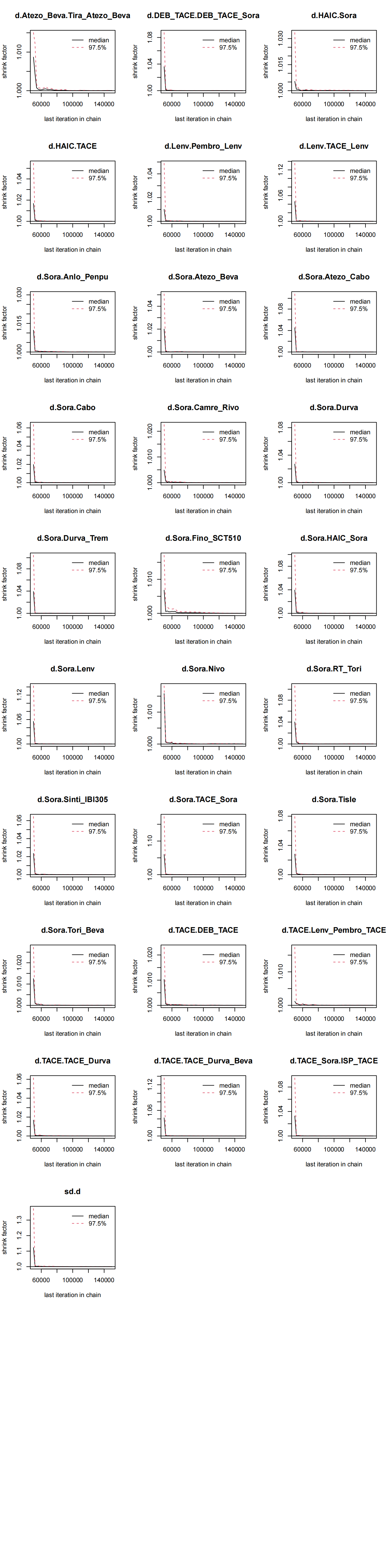** \| \| --- \| \| **Figure S6. Convergence Diagnostics of Bayesian Network Meta-Analysis for AEs≥3** \| |
| --- | --- | --- |

**
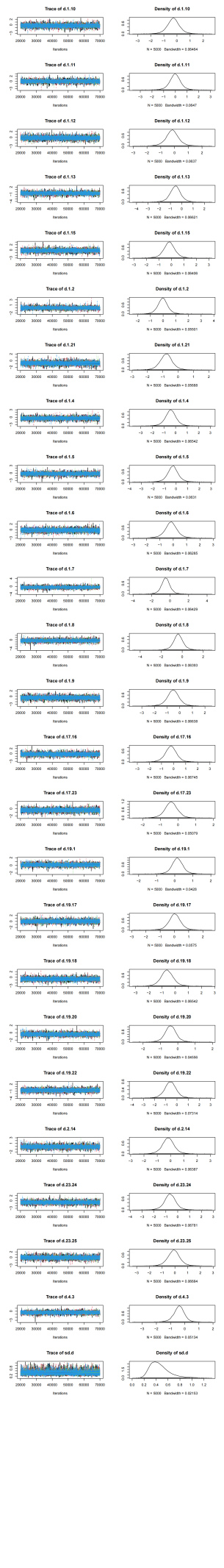
**

**Figure S7. Trace and Posterior Density Plots for Bayesian Network Meta-Analysis of OS**

**
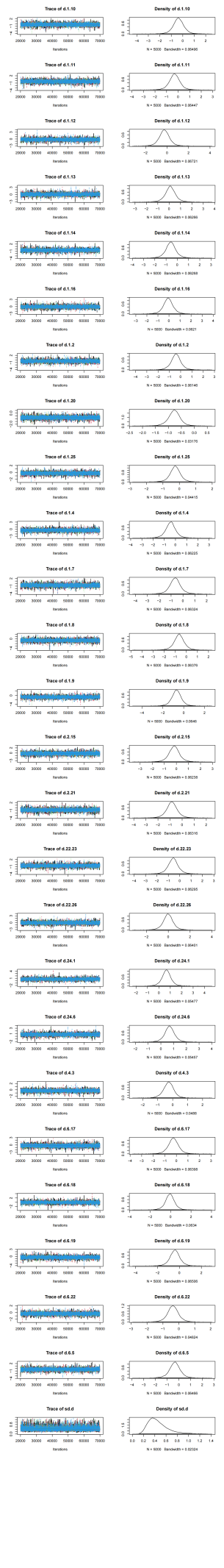
**

**Figure S8. Trace and Posterior Density Plots for Bayesian Network Meta-Analysis of PFS**

**
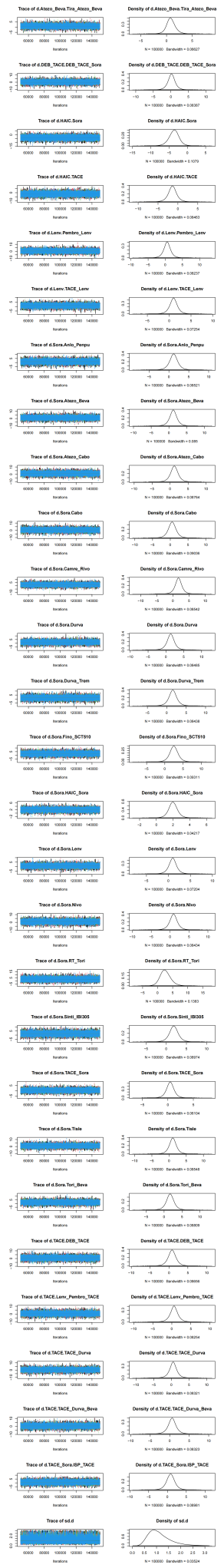
**

**Figure S9. Trace and Posterior Density Plots for Bayesian Network Meta-Analysis of ORR**

**
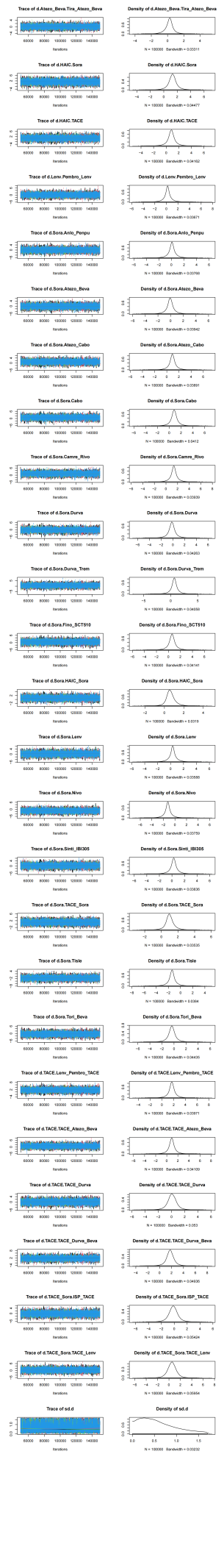
**

**Figure S10. Trace and Posterior Density Plots for Bayesian Network Meta-Analysis of AEs≥3**

**
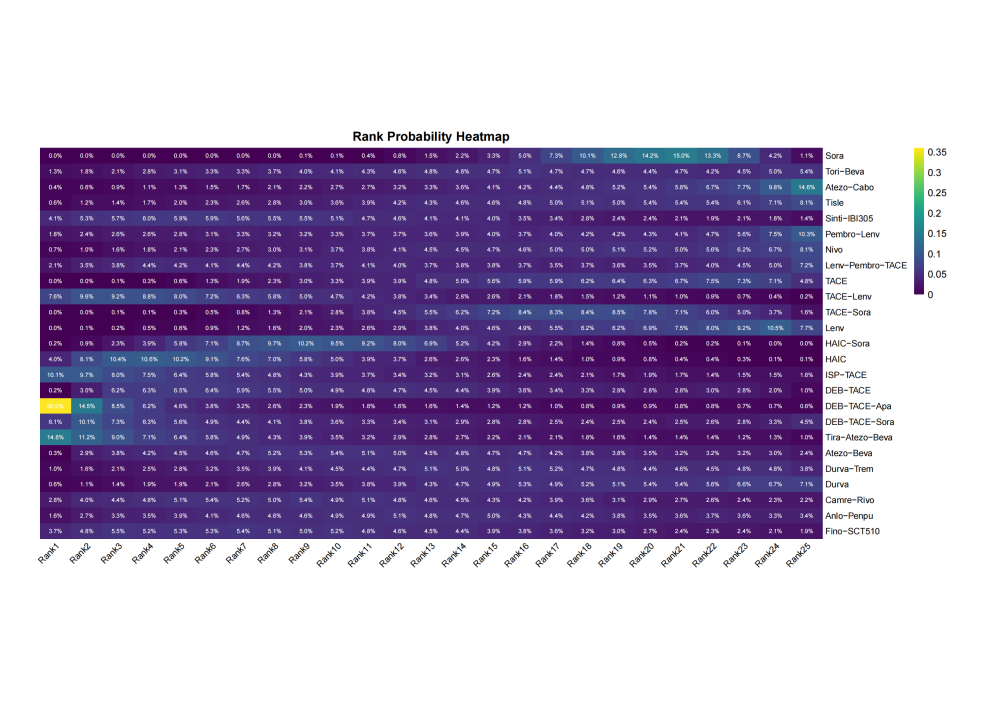
**

**Figure S11. Rank Probability Heatmap of Treatment Regimens for OS**

**
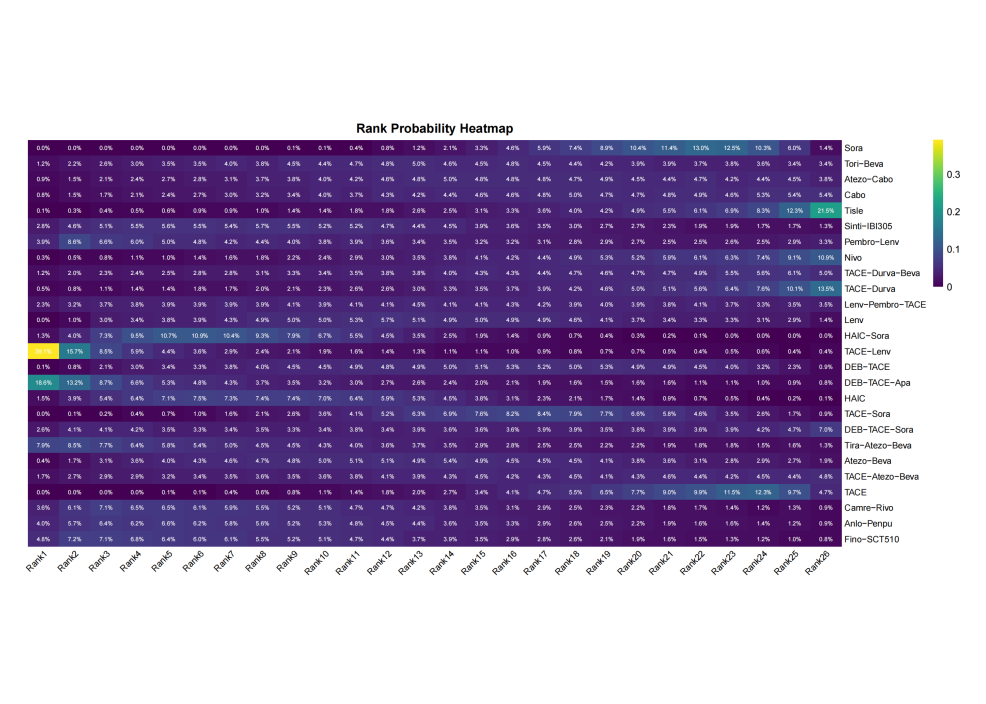
**

**Figure S12. Rank Probability Heatmap of Treatment Regimens for PFS**

**
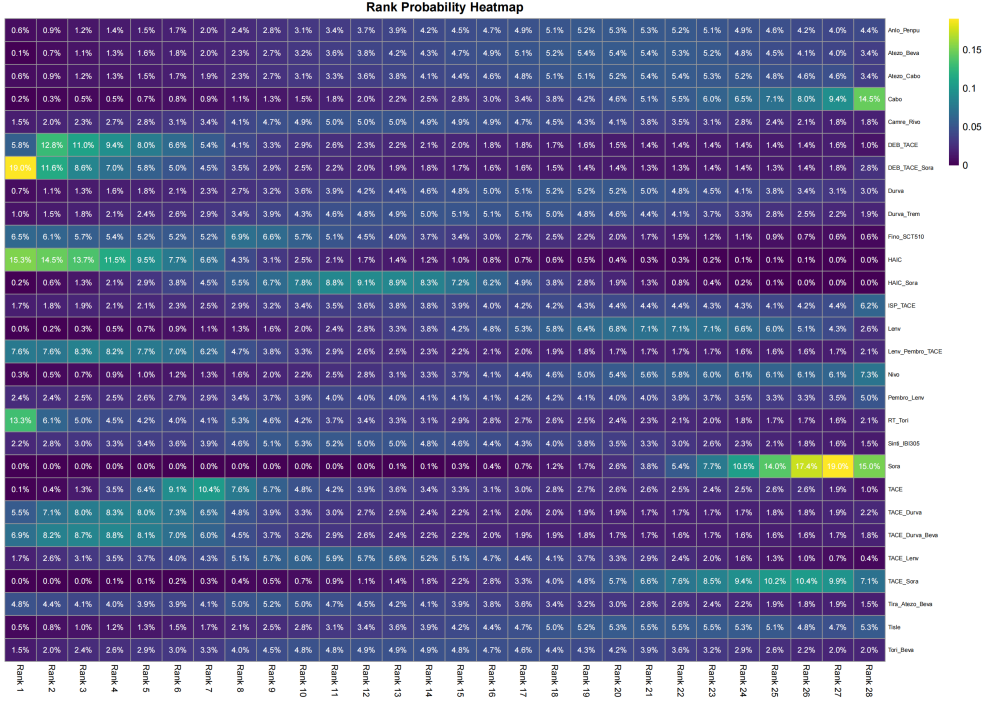
**

**Figure S13. Rank Probability Heatmap of Treatment Regimens for ORR**

**
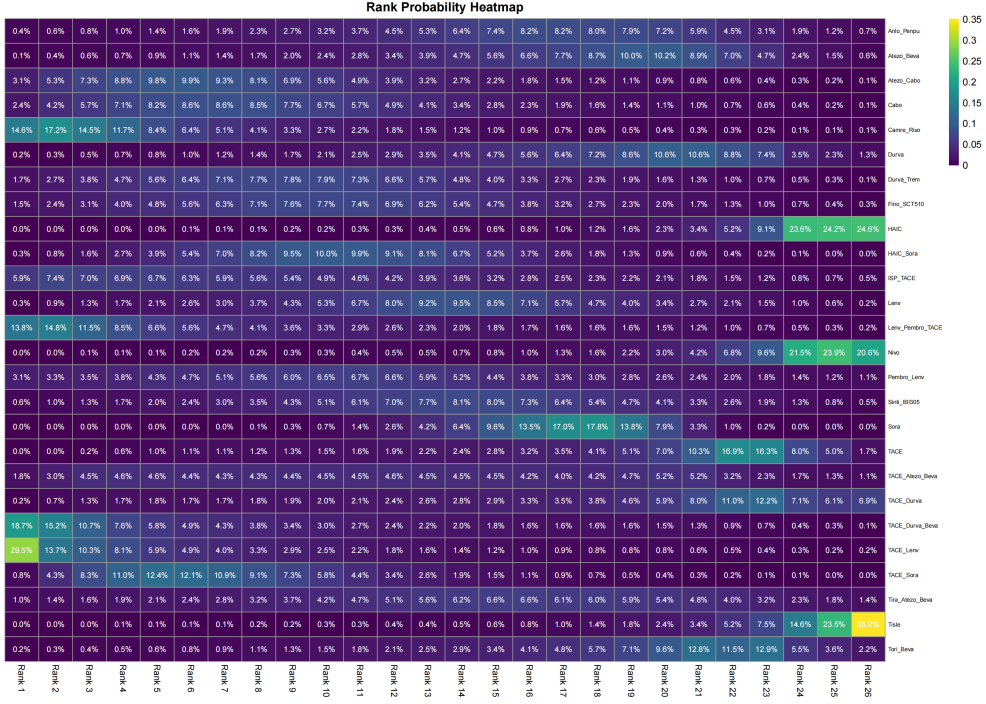
**

**Figure S14. Rank Probability Heatmap of Treatment Regimens for AEs≥3**

**
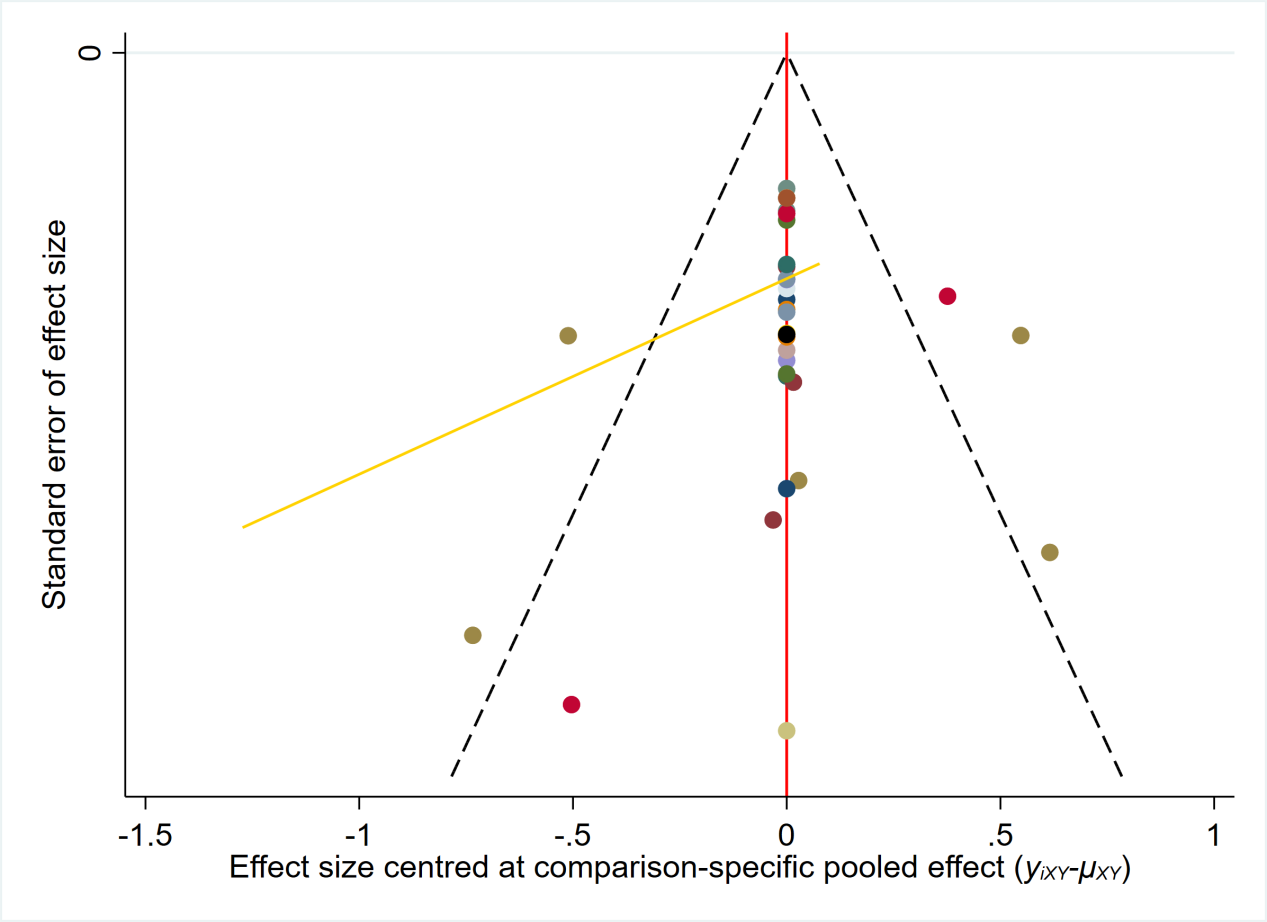
**

**Figure S15. Comparison-adjusted Funnel Plot for OS**

**
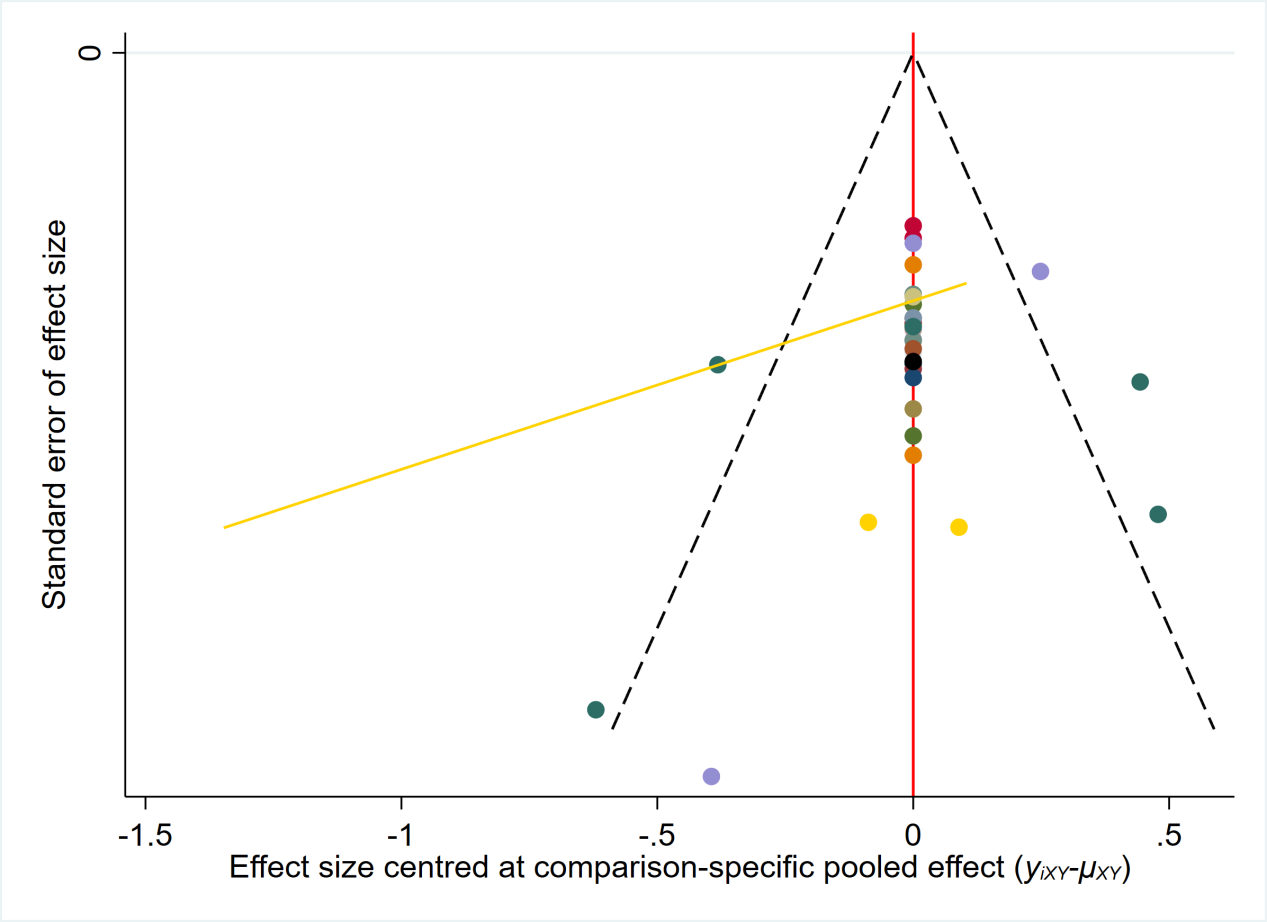
**

**Figure S16. Comparison-adjusted Funnel Plot for PFS**

**
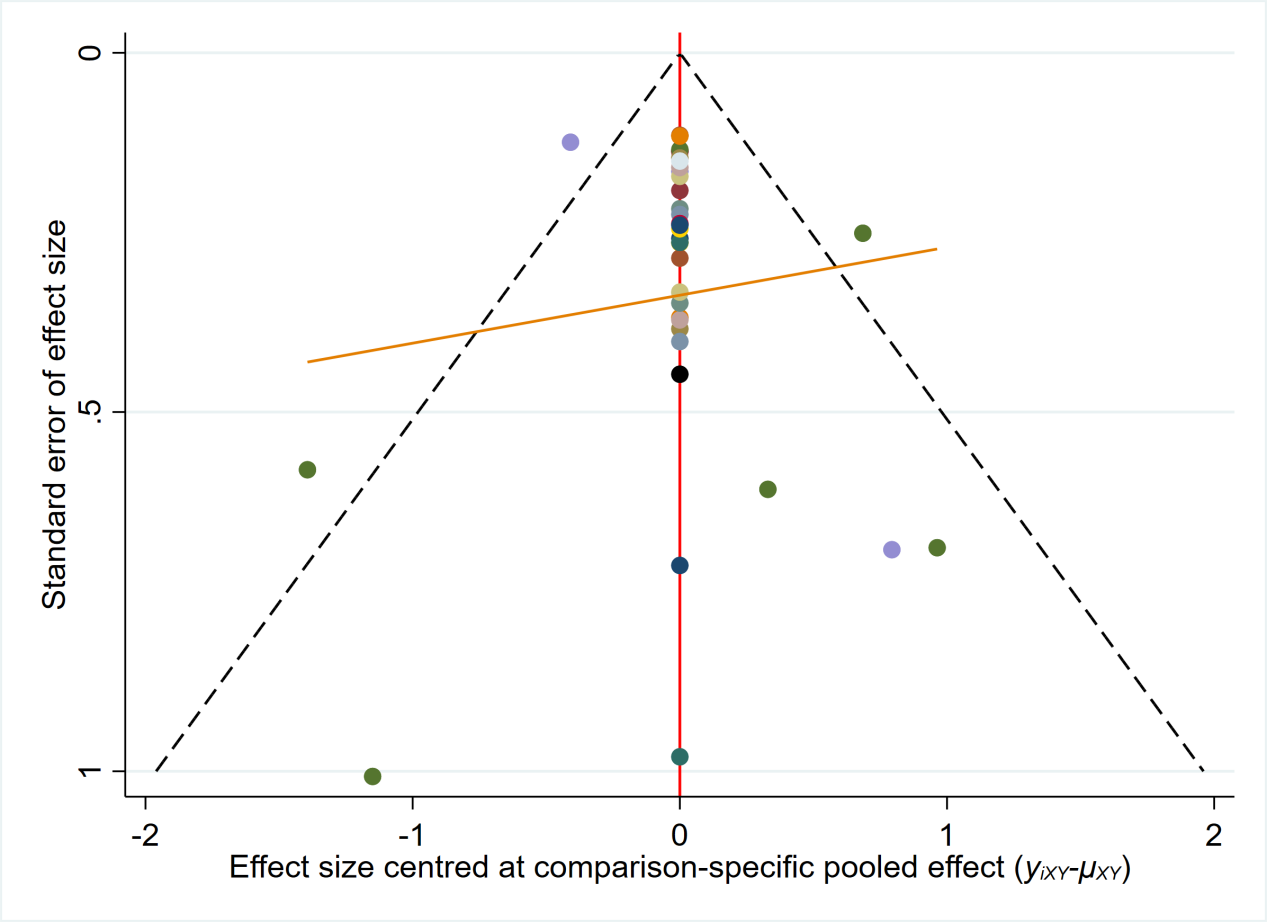
**

**Figure S17. Comparison-adjusted Funnel Plot for ORR**

**
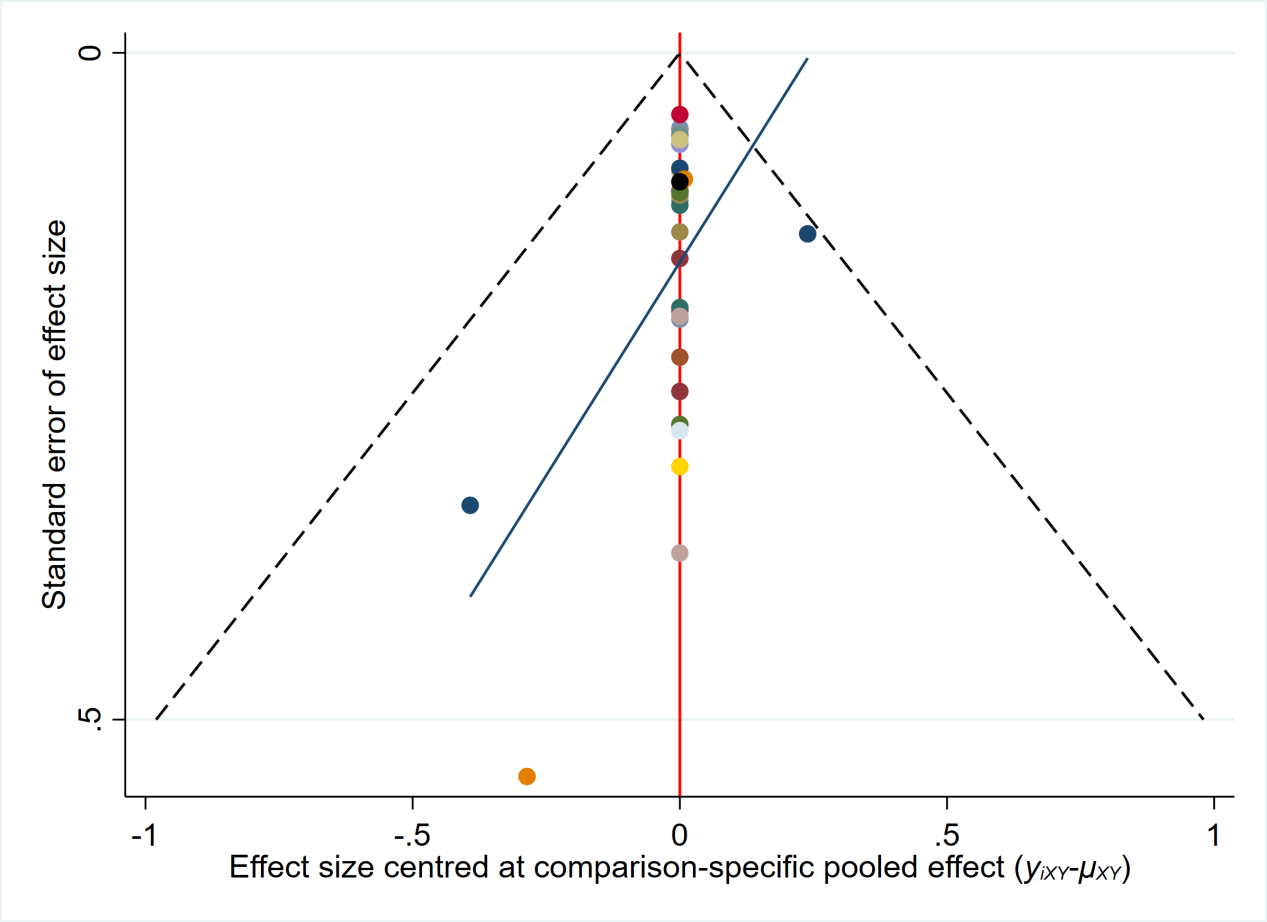
**

**Figure S18. Comparison-adjusted Funnel Plot for AEs≥3**
